# The importance of fusion protein activity in Ewing sarcoma and the cell intrinsic and extrinsic factors that regulate it: A review

**DOI:** 10.3389/fonc.2022.1044707

**Published:** 2022-11-24

**Authors:** April A. Apfelbaum, Emma D. Wrenn, Elizabeth R. Lawlor

**Affiliations:** Ben Towne Center for Childhood Cancer Research, Seattle Children's Research Institute and Department of Pediatrics, University of Washington, Seattle, WA, United States

**Keywords:** Ewing sarcoma, heterogeneity, plasticity, oncogene, cell phenotype

## Abstract

Accumulating evidence shows that despite clonal origins tumors eventually become complex communities comprised of phenotypically distinct cell subpopulations. This heterogeneity arises from both tumor cell intrinsic programs and signals from spatially and temporally dynamic microenvironments. While pediatric cancers usually lack the mutational burden of adult cancers, they still exhibit high levels of cellular heterogeneity that are largely mediated by epigenetic mechanisms. Ewing sarcomas are aggressive bone and soft tissue malignancies with peak incidence in adolescence and the prognosis for patients with relapsed and metastatic disease is dismal. Ewing sarcomas are driven by a single pathognomonic fusion between a FET protein and an ETS family transcription factor, the most common of which is EWS::FLI1. Despite sharing a single driver mutation, Ewing sarcoma cells demonstrate a high degree of transcriptional heterogeneity both between and within tumors. Recent studies have identified differential fusion protein activity as a key source of this heterogeneity which leads to profoundly different cellular phenotypes. Paradoxically, increased invasive and metastatic potential is associated with *lower* EWS::FLI1 activity. Here, we review what is currently understood about EWS::FLI1 activity, the cell autonomous and tumor microenvironmental factors that regulate it, and the downstream consequences of these activity states on tumor progression. We specifically highlight how transcription factor regulation, signaling pathway modulation, and the extracellular matrix intersect to create a complex network of tumor cell phenotypes. We propose that elucidation of the mechanisms by which these essential elements interact will enable the development of novel therapeutic approaches that are designed to target this complexity and ultimately improve patient outcomes.

## Introduction

Intratumoral heterogeneity has been identified as a key factor contributing to therapeutic failure and emerging drug resistance across cancer types (1–3). While it has long been appreciated that genomic instability underlies genetic heterogeneity, more recent studies have implicated non-mutational epigenetic heterogeneity in driving tumor progression and resistance (4). Pediatric tumors have low mutational burden; however, they exhibit epigenetic heterogeneity including altered expression and activity of transcriptional regulators, chromatin remodelers, and developmental programs which collectively regulate transcription and cell state (5). In many cases these different tumor cell states reflect different states of normal developmental trajectories (6). The epigenetic heterogeneity and plasticity of pediatric solid tumors makes them challenging to eradicate, particularly in a manner that spares normal developing tissues which engage, and are dependent on, parallel biologic processes.

Ewing sarcomas are prototypical examples of this dynamic. Despite being driven by a single, shared genetic mutation (discussed in detail below), they are epigenetically very plastic tumors and present considerable clinical heterogeneity. Ewing sarcomas present across a wide range of ages, from young children to elderly adults, but peak incidence is in older children, adolescents, and young adults. The tumors most commonly present in the bones such as the pelvis and long bones, however 20% of cases arise in extraosseous sites (7, 8). For localized tumors, multi-agent neoadjuvant and adjuvant chemotherapy combined with surgical resection and/or radiotherapy for local control can cure over 70% of patients (7, 8). However, survivors experience high morbidity and reduced life expectancy due to long-term side effects and secondary malignancies caused by treatment (9). Survival rates for patients who present or relapse with metastatic disease are less than 30% (10). This disparity highlights a critical need to understand the molecular mechanisms underlying Ewing sarcoma treatment resistance and metastasis.

Ewing sarcomas are driven by pathognomonic fusion proteins that arise from chromosomal translocations between FET and ETS family protein-encoding genes. These fusion proteins are ubiquitously expressed under the FET family promoter and encode aberrant transcription factors that cause transcriptional dysregulation. EWS::FLI1 was the first identified fusion protein and occurs in ~85% of tumors (7, 11). Ten percent of tumors express EWS::ERG and the remainder harbor more rare variants (reviewed in (7, 12)). EWS::ETS fusions play diverse roles in regulating many molecular processes including chromatin architecture, gene transcription, RNA splicing, R-loop formation, and protein translation (Reviewed in (7, 8)). Additional recurrent mutations are rare but mutation(s) in *TP53 (7-9%)*, *CDKN2A (10-22%)*, or *STAG2 (15-21%)*, are reported to be associated with more aggressive disease (7, 8, 13, 14). To date, little is known about the contribution of clonal evolution to Ewing sarcoma progression but limited studies of paired patient biopsies from primary and metastatic or relapsed sites suggest that, unlike many adult cancers, emergence and outgrowth of subclonal disease does not drive progression (2, 13, 15). Rather, disease progression and tumor heterogeneity in Ewing sarcoma appear to be driven by epigenetic mechanisms that enable phenotypic plasticity (4, 16).

Over the past several years, independent findings from multiple labs have shown that the levels of EWS::FLI1 expression and transcriptional activity vary among individual tumor cells and this variability is emerging as a critical determinant of epigenetic plasticity, tumor cell phenotype, and disease progression (17–25). Here, we will review the current state of knowledge in the field regarding EWS::FLI1 fusion activity and the tumor cell intrinsic and extrinsic factors that regulate it. We will discuss how different fusion activity states might influence tumor progression and treatment response and how crosstalk with the tumor microenvironment serves as a critical regulator of both fusion activity and tumor cell behavior.

## Characteristics of the EWS::FLI1 “high” and “low” cell states

**Figure 1:**
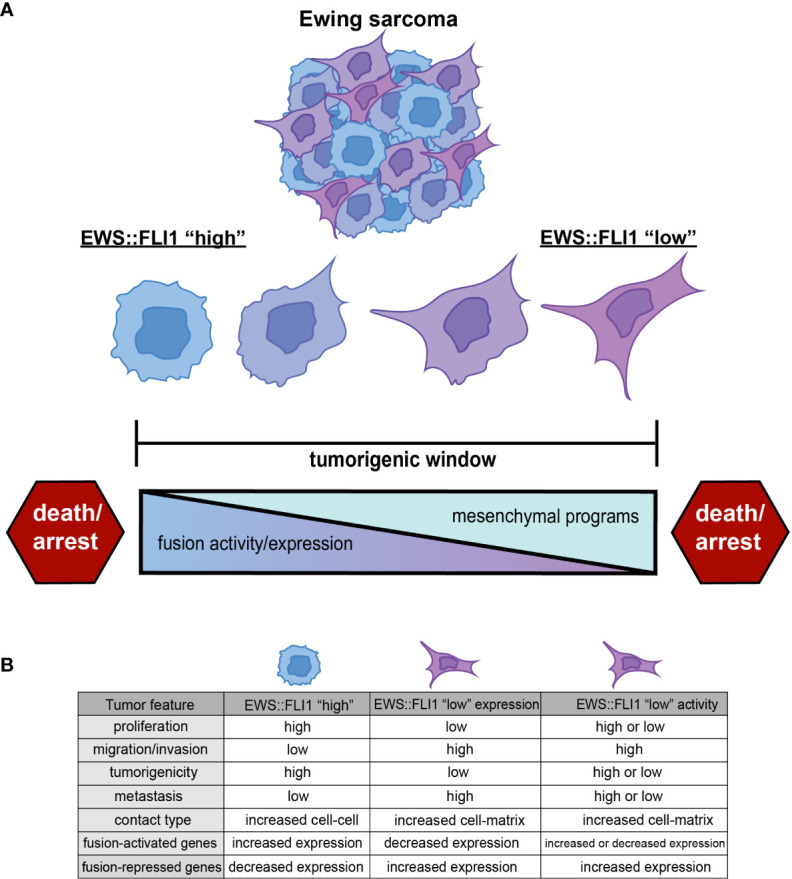
**(A)** Ewing sarcoma cells exist along a spectrum of EWS::FLI1 expression and activity states. Ewing tumors consist of different populations of these cells, and epigenetic plasticity allows cells to shift along this axis within the tumorigenic window. EWS::FLI1 activity above or below a permissive window leads to growth arrest and death, consistent with the “Goldilocks” principle (19). **(B)** EWS::FLI1 “high” cells have increased proliferation, tumorigenic potential, and exhibit more cell-cell contact. EWS::FLI1 “low” cells have increased migration/invasion, metastatic potential, and enhanced extracellular matrix association (17–25). EWS::FLI1 “high” and “low” cells have an inverse expression pattern of fusion-regulated genes. EWS::FLI1 “low” activity cells have increased expression of the repressed signature, but can have either high or low expression of the activating signature. Figure created with BioRender.com.

EWS::FLI1 promotes tumorigenesis, cell proliferation, and tumor expansion (7). However, EWS::FLI1-dependent transformation and tumorigenicity depend on the level of expression and activity of the fusion oncogene. In non-permissive cell types, or when expressed too highly in Ewing sarcoma or permissive progenitor cells, EWS::FLI1 induces cell cycle arrest and death (19, 26–28). This has led to the premise that Ewing sarcomas are subject to the “Goldilocks” principle in which EWS::FLI1 must be maintained at just-right levels: too much fusion protein is toxic, while too little fails to maintain malignant properties (19) (**Figure 1A**).

The first clues that levels of fusion expression might contribute to cell plasticity and tumor behavior came from early studies of EWS::FLI1 knockdown. Reduction of EWS::FLI1 expression leads to changes in the Ewing sarcoma cell cytoskeleton that resulted in a larger and more spindle-like morphology that is associated with altered adhesive properties (17, 18, 29–31). Fusion-dependent regulation of YAP signaling has since been identified as a downstream mediator of these cytoskeletal changes (24, 32). In addition, EWS::FLI1 knockdown cells show increased Rho pathway activation and higher mesenchymal-identity gene expression (17, 24, 29, 30). These transcriptional and phenotypic changes are associated with promotion of cell migration, invasion, and metastatic fitness.

Over the past few years, advances in genomic technologies have allowed for assessment of heterogeneity in endogenous EWS::FLI1 expression and activity in tumors in situ, rather than relying exclusively on EWS::FLI1 knockdown and overexpression models. These studies have shown that expression and transcriptional activity of the fusion varies among tumor cells and that cells exist along a transcriptional continuum from less to more mesenchymal states. Significantly, this heterogeneity is evident in tumor-derived cell lines and patient-derived xenografts (PDX) *in vitro* and *in vivo* and in patient tumor biopsies (17, 22, 33, 34). Consistent with genetic knockdown studies, cells with higher EWS::FLI1 activity express proliferative signatures, while cells with lower transcriptional activity upregulate mesenchymal gene signatures and display enhanced metastatic potential (17, 22, 33). Elucidating the contribution of these distinct cell states to local and metastatic progression, treatment response, and relapse is an area of intense investigation in the field.

Throughout this review, we will use the term EWS::FLI1 “high” to refer to cells in which EWS::FLI1 is both highly expressed and transcriptionally active. We will use the term EWS::FLI1 “low” to refer to cells in which the transcriptional activity of EWS::FLI has been inhibited in some way. EWS::FLI1 “high” cells make up the bulk of Ewing sarcoma cells and are, in general, proliferative and relatively immotile. In contrast, EWS::FLI1 “low” cells exist as minority subpopulations, display more mesenchymal and migratory phenotypes, and have been implicated in promoting metastasis. These fusion “low” cells can arise *via* depletion of the EWS::FLI1 protein itself or by inhibition of its function as a transcriptional activator and/or repressor (**Figure 1B**). Although these definitions of EWS::FLI1 “high” and “low” states are a simplification and do not adequately convey the continuum of fusion protein activity that is observed in Ewing sarcoma cells, they serve as paradigm for this review and as a starting point from which the field can build.

## Distinct mechanisms of gene activation and repression by EWS::FLI1

The EWS::FLI1 “high” and “low” states are transcriptionally defined by the relative expression of fusion target genes. Reduction of the fusion leads to decreased expression of EWS::FLI1-activated targets and increased expression of repressed targets. However, the mechanisms by which the fusion activates and represses target genes are distinct and, although significant gaps in knowledge still exist, it is evident that the activation and repressive signatures can be dissociated depending on the presence of additional regulators of these separate processes (20–22). Below we summarize the current understanding of how EWS::FLI1 alters gene transcription.

### EWS::FLI1 activates genes via GGAA enhancer reprogramming

EWS::FLI1 has both transcriptional activating and repressive functions, which are both critical for successful oncogenesis (35). One of its most well studied roles in gene activation is as an aberrant transcription factor that rewires the epigenome through enhancer reprogramming. EWS::FLI1 acts as a pioneer factor to generate active *de novo* enhancers by increasing chromatin accessibility, directing recruitment of histone acetyl/methyltransferases, and establishing long-range interactions at GGAA microsatellites (36, 37). These GGAA enhancer sites act as distal regulatory elements to specific gene targets uniquely upregulated in Ewing sarcoma (38). Many EWS::FLI1-bound GGAA sites are usually epigenetically silent and not evolutionarily conserved, suggesting a limited role in normal transcriptional programs (36). This lack of GGAA conservation likely contributes to difficulty generating representative animal models of Ewing sarcoma (39). A subset of EWS::FLI1-activated genes are critical for oncogenesis and many induce proliferation (40, 41). The EWS::FLI1-activated signature is also heterogeneous because humans exhibit GGAA microsatellite polymorphisms which can correlate with disease susceptibility (42, 43). These polymorphisms may underlie discrepancies and variation in expression of distinct target genes across different Ewing sarcoma cell lines (44, 45). While most of the current understanding of fusion-dependent gene activation is centered around GGAA repeat microsatellites, EWS::FLI1 can also directly activate target gene expression by binding to non-GGAA repeat sites, primarily wild-type ETS binding sites consisting of shorter GGAA motifs (46). One study estimated that 25% of EWS::FLI1 binding sites are at active cis-regulatory non-GGAA repeats (36). Understanding how the GGAA and non-GGAA repeat landscape influences and alters the EWS::FLI1 activation signature will be important for future investigations of inter-tumoral heterogeneity.

### EWS::FLI1 mediated gene repression

While the mechanisms of EWS::FLI1-dependent gene activation have been widely studied, the mechanisms of transcriptional repression remain less clear but both direct and indirect mechanisms have been described. EWS::FLI1 can directly repress transcription by binding to wild-type ETS family binding sites in gene promoters and enhancers. These sites typically have a single ETS consensus sequence or a small number of GGAA repeats and EWS::FLI1 binding displaces the more potent wild-type transcriptional activator resulting in downregulation of gene transcription (35, 36). Indirect mechanisms of EWS::FLI1-mediated gene repression rely on direct activation of transcriptional repressors such as NKX2-2 (30, 47) and the lncRNA EWSAT1 (48), as well as fusion-driven recruitment of repressive epigenetic proteins and complexes including HDACs (47), LSD1 and the NuRD complex (46, 49) to target loci. The combined effect of these direct and indirect mechanisms results in downregulation of hundreds of genes, many of which regulate mesenchymal identity (37, 50). Significantly, derepression of this mesenchymal signature is associated with acquisition of metastatic properties. The Ewing sarcoma cell(s) of origin are imprecisely defined.

## The impact of cell of origin on tumor cell heterogeneity

While the exact Ewing sarcoma cell or cells of origin is still an enigma, studies have shown that both neural crest stem cells (NCSCs) and mesenchymal stem cells (MSCs) tolerate EWS::FLI1 expression and lead to increased Ewing-like gene expression and morphology (51–54). Knockdown of EWS::FLI1 expression in Ewing sarcoma cells induces transcriptomes that closely resemble MSCs (18, 50, 55). More recently, a large-scale study which reconstructed extensive transcriptomic data from tumor and normal tissues revealed that Ewing cells have signatures of early developmental lineages distinct from post-natal MSCs, specifically mesoderm and pluripotent/neuroectodermal cell types and that tumors and cells exist along a transcriptional spectrum (56). In addition, DNA methylation is increasingly being used to map tumors to cells of origin and a study of DNA methylation in Ewing sarcoma tumors identified profound intra- and inter-tumoral heterogeneity with respect to DNA methylation profiles (57). This study also defined the existence of tumor cells across a spectrum of mesenchymal to stem cell states, both between and within tumors. Whether this epigenetic heterogeneity influences or is influenced by EWS::FLI1 activity is as yet unclear.

An attractive hypothesis may be that intratumoral heterogeneity in Ewing sarcoma and differences in EWS::FLI1 activity arise due to distinct cells of origin, or transformation of the same cell of origin but at different developmental time points or contexts. The cell of origin cannot yet be conclusively determined retrospectively from patient samples and Ewing sarcoma mouse models that faithfully model disease initiation do not exist, possibly due to species-specific enhancer functions of EWS::FLI1 (39). Still, despite lasting ambiguity of the exact cell of origin, increasing evidence suggests that Ewing sarcoma cells and tumors exist along a quasi-neuroectodermal vs. mesenchymal cell state spectrum. It is thus likely that intrinsic differences in the chromatin state, DNA methylation profile, and transcription factor repertoire of the cell of origin influence EWS::FLI1 activity, cell state, and tumor behavior.

## Regulation of EWS::FLI1 expression and activity

The effects of EWS::FLI1 on target gene expression are determined by both the absolute level of fusion protein expression and by complex interactions with cell autonomous and cell extrinsic factors that influence its activity as a transcriptional activator or repressor. Here we review what is known about these factors in this rapidly evolving area of inquiry. As new findings emerge about the contribution of tumor cell heterogeneity to tumor progression, drug resistance, and metastasis, a deeper understanding of the features that regulate EWS::FLI1 activity may inform the development of targeted therapies that can moderate them.

## Cell autonomous regulators of EWS::FLI1 transcript expression

Regulation of the level of EWS::FLI1 fusion protein in Ewing sarcoma cell begins with activation of the *EWSR1* promoter, which serves as the promoter of the fusion gene (8). Unlike the FLI1 protein, wild-type EWS protein is ubiquitously expressed. Though little is known about *EWSR1* transcriptional regulation, a recent study found that HDAC6 inhibition suppressed *EWS::FLI1* transcription by modulating binding of the SP1/P300 complex at the *EWSR1* promoter region (58). This demonstrates that transcription of the fusion gene is at least partially under epigenetic control. Levels of fusion gene transcript expression are also controlled by mRNA splicing and processing. In particular, the splicing factor SF3B1 and mRNA processing protein HNRNPH1 regulate EWS::FLI1 mRNA (59). Finally, EWS::FLI1 mRNA stability contributes to fusion expression. A minority of Ewing sarcomas express LIN28B which can directly bind EWS::FLI1 transcripts, promoting their stability (60).

**Figure 2:**
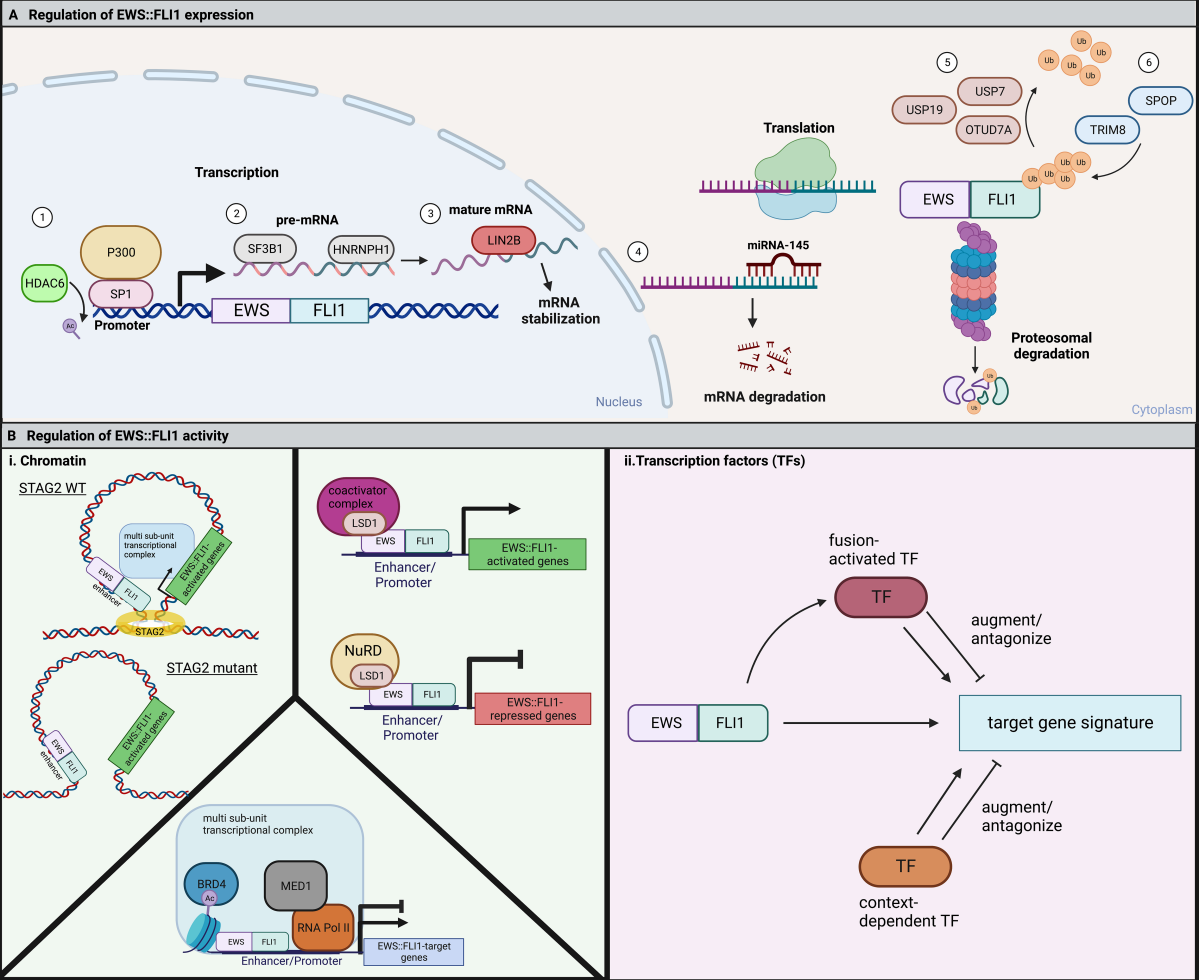
**(A)** EWS::FLI1 expression and activity are highly regulated through many cell-intrinsic processes. 1) The EWS::FLI1 and endogenous EWSR1 promoter are positively regulated by SP1 in complex with the histone acetyltransferase, P300. HDAC6 keeps SP1 deacetylated, allowing for continued binding and expression of the fusion (58). 2) SF3B1 is a member of the spliceosome that is critical to for proper pre-mRNA splicing of EWS::FLI1. HNRNPH1 is a RNA binding protein that facilitates the splicing of EWSR1 exon 8-containing fusions (59). 3) The RNA-binding protein LIN28B can bind to and stabilize EWS::FLI1 mRNA (60). 4) In opposition, the microRNA miRNA-145 can bind the 3’ UTR of FLI1 and cause mRNA degradation of the fusion (64). EWS::FLI1 protein is degraded by the proteasome and regulated by the competing action of deubiquitinases and E3 ubiquitin ligases. 5) USP7, USP19, and OTUD7A are three deubiquitinases that have been shown to promote EWS::FLI1 expression (67–69). 6) TRIM8 and SPOP are two E3 ubiquitin ligases that have been reported to promote EWS::FLI1 degradation (19, 69). **(B)** (i) EWS::FLI1 activity is regulated by chromatin topologies and chromatin modifying complexes. In Ewing sarcoma cells with mutant STAG2, the EWS::FLI1 transcriptional signature is disrupted by alterations in chromatin looping (20, 21). LSD1 functions to regulate fusion activity in Ewing sarcoma cells through interaction with the fusion itself and either coactivator or repressive (NuRD) complexes resulting in either gene activation or repression (35, 49, 70–72). BRD4 regulates both the fusions activating and repressive activity through indirect binding with EWS::FLI1 in a large multi-subunit transcriptional complex (73). ii. EWS::FLI1 activity is also regulated by fusion-activated TFs or context-dependent TFs. These TFs then can either augment or antagonize expression of different subsets of the EWS::FLI1 target gene signature. Figure created with BioRender.com.

Non-coding RNAs (ncRNAs), including miRNAs and lncRNAs, are key regulators of transcription, translation, and signaling pathways. There is evidence that these ncRNAs are important epigenetic and transcriptional regulators of Ewing sarcoma oncogenesis (61, 62). In addition, while EWS::FLI1 can modulate ncRNA expression, the fusion may in turn be regulated by these transcripts (48, 63). For example, miRNA-145 is upregulated upon knockdown of EWS::FLI1, and itself reduces EWS::FLI1 expression by binding to the *FLI1* 3’-untranslated region (64). This feedback loop acts as a cell autonomous regulator of fusion gene expression and activity. Accordingly, overexpression of miRNA-145 leads to downregulation of EWS::FLI1 and an increase in mesenchymal gene expression (51). While primary data are limited, it is likely that the pleiotropic effects of EWS::FLI1 on expression of multiple ncRNAs may impact on stability or translation of the fusion transcript and thereby influence its level of expression (65, 66) (**Figure 2A**).

### EWS::FLI1 protein is subject to proteasomal degradation

After translation, EWS::FLI1 levels are regulated by proteasomal degradation (**Figure 2A**). The half-life of EWS::FLI1 protein (1-4 hours) is shorter than either wild-type EWS or FLI1 (4 to 24 hours), suggesting that the fusion protein is highly sensitive to degradation (74). Three deubiquitinases (USP7 (67), USP19 (68), and OTUD7A (69)) are required to prevent proteasomal degradation of the fusion. Reduction of any of these enzymes leads to reduced levels of the fusion and impedance of Ewing sarcoma cell viability and growth. Conversely, loss of ubiquitin ligases also impedes tumorigenicity. TRIM8 was identified in the Cancer DepMap project as a unique dependency in Ewing sarcoma cells relative to any other cell type and EWS::FLI1 was subsequently confirmed to be a neomorphic substrate of this E3 ligase (19, 75). Loss of TRIM8 leads to an increase in EWS::FLI1 protein and apoptosis due to oncogene overdose (19). The E3 ligase, Speckle Type BTB/POZ Protein (SPOP) also plays a key role in regulating the half-life of EWS::FLI1 and, along with its corresponding deubiquitinase OTUD7A, serves to maintain balanced protein expression (69). These data together reveal a key role for post-translational regulation in maintaining “just right” levels of the fusion. It remains to be elucidated if and how dynamic regulation of these ubiquitin ligases and deubiquitinases contributes to cell plasticity and tumor cell heterogeneity.

### Epigenetic factors that influence the EWS::FLI1-dependent transcriptome

To enact transcriptional regulation EWS::FLI1 interacts with multiple chromatin remodeling complexes and the transcriptional machinery (**Figure 2B**). The ability of these proteins to modify transcription depends on the existing three-dimensional structure of the genome and its segregation into topologically-associated domains (TADs). *STAG2* is among the few genes that are recurrently mutated in Ewing sarcoma, occurring in approximately 17% of patients and correlating with poorer prognosis (13, 14, 76). STAG2 is a subunit of the cohesin complex which helps control sister chromatid alignment and define the boundaries of TADs. STAG2 also regulates intrachromosomal promoter-enhancer interactions. Interestingly, loss of STAG2 in Ewing sarcoma cells results in impaired loop extrusion and alters promoter-enhancer interactions which impedes the EWS::FLI1-dependent transcriptional program. Notably, loss of STAG2 does not alter EWS::FLI1 expression itself, but rather alters its chromatin distribution at distinct target genes (20, 21). Most prominently, pro-metastatic, mesenchymal genes are de-repressed in STAG2 mutant cells, which also have metastatic properties consistent with an EWS::FLI1 “low” state (20, 21). As discussed above, cell intrinsic and extrinsic factors that impact on EWS::FLI1 activity often influence expression of only discrete sets of target genes rather than the entire fusion-responsive gene signature. Dissociation of transcriptional activating and repressive functions of the fusion has the potential to fine tune cell phenotypes beyond simple “high” and “low” states.

Other chromatin remodelers cooperate with EWS::FLI1 to maintain oncogenic gene expression programs and tumorigenic cell states. Lysine-specific demethylase 1 (LSD1), a H3K4/9 demethylase, is recruited with the NuRD complex to gene promoters to aid in fusion-dependent gene repression (35, 49, 70–72). Newer large-scale genomics studies have revealed that LSD1 also plays a critical role in EWS::FLI1-dependent gene activation, binding alongside the fusion at both GGAA microsatellites and non-GGAA microsatellite binding sites (46). Bromodomain-containing protein 4 (BRD4), an epigenetic reader that regulates gene expression through recognition of acetylated histones, indirectly interacts with EWS::FLI1 to regulate expression of fusion-activated and fusion-repressed target genes (73). Genetic and pharmacologic inhibition of BRD4 antagonizes the EWS::FLI1 target gene signature and inhibits oncogenic cell phenotypes (73). KDM3A (a H3K9me1/2 demethylase) is highly overexpressed by Ewing sarcoma cells and promotes pro-metastatic and migratory gene expression (77, 78). KDM3A is indirectly positively regulated by EWS::FLI1 and negatively regulated by miRNA-22, which is repressed by EWS::FLI1. EWS::FLI1 repression of miRNA-22 allows for overexpression of KDM3A, which selectively activates genes critical for the metastatic process. Together these studies reveal the critical role of cell intrinsic epigenetic programs in modulating the transcriptional activity of EWS::FLI1 and further demonstrate that activating and repressive properties can be regulated separately and locally.

### Transcription factors can promote or attenuate EWS::FLI1-dependent gene expression

For successful epigenetic and transcriptional reprogramming, EWS::FLI1 alters the expression of multiple transcription factors (TFs) which then cooperate with or antagonize the fusion and its transcriptional activity (**Figure 2B** and **Table 1**). These secondary TF programs play critical roles in both initiation and maintenance of Ewing sarcoma tumorigenicity, including through regulation of EWS::FLI1 itself. Some TFs activated by EWS::FLI1 act cooperatively to upregulate fusion target genes. For example, the homeobox TF, MEIS1, co-binds with EWS::FLI1 at 25% of binding sites and augments expression of a subset of EWS::FLI1-activated genes (79). Another developmental TF, RUNX3, can directly interact with EWS::FLI1 and coordinately regulate a subset of activated and repressed target genes (80). For FEZF1, a TF involved in nervous system development, 38% of FEZF1-regulated genes are also EWS::FLI1 target genes (81). In addition, EWS::FLI1 directly activates E2F family TFs, specifically E2F3, which positively regulate EWS::FLI1-activated genes and promote proliferation (82, 83). These EWS::FLI1-activated transcription factors can also cooperate with one another. One recent study found that NKX2-2, TCF4, and KLF15 all positively regulate each other by promoter and super-enhancer binding, and cooperatively upregulate EWS::FLI1-activated targets (84). These EWS::FLI1-upregulated TFs greatly expand the number of gene targets that are deregulated by EWS::FLI1 and in some cases they are also required for efficient tumor outgrowth (79, 84, 85).

**Table 1:** Description of a select subset of transcription factors that are either regulated by EWS::FLI1 or play an important role in altering expression of EWS::FLI1 targets.

TF	Regulated by EWS::FLI1?	Direct EWS::FLI1 target?	Effect on EWS::FLI1	Effect on EWS::FLI1 targets
MEIS1 (79)	Upregulated by EWS::FLI1	NR	NR	Augments EWS::FLI1 activated signature
RUNX3 (80)	EWS::FLI1 inhibits RUNX3 activity	NR	Binds EWS::FLI1	De-represses genes downregulated by EWS::FLI1
FEZF1 (81)	Upregulated by EWS::FLI1	Yes	No effect on EWS::FLI1 expression	Augments EWS::FLI1 signature, particularly neural genes
E2F3 (82, 83)	Upregulated by EWS::FLI1	NR	NR	Augments EWS::FLI1 signature
NKX2-2 (30, 47, 79)	Upregulated by EWS::FLI1	Yes	No effect on EWS::FLI1 expression	Augments EWS::FLI1 repressed signature
HOXD13 (22)	Upregulated by EWS::FLI1	Yes	No effect on EWS::FLI1 expression	De-represses genes downregulated by EWS::FLI1
ZEB2 (30, 90)	Expression not regulated by EWS::FLI1	No	No effect on EWS::FLI1 expression	De-represses genes downregulated by EWS::FLI1
NR0B1 (25, 91, 172)	Upregulated by EWS::FLI1	Yes	Binds EWS::FLI1	Augments EWS::FLI1 signature
BCL11B (92, 173)	Upregulated by EWS::FLI1	Yes	NR	Augments EWS::FLI1 repressed signature
GLI1 (83, 93, 174)	Upregulated by EWS::FLI1	Yes	NR	Augments EWS::FLI1 signature
SOX2 (36, 38, 51)	Upregulated by EWS::FLI1	Yes, and regulated by miRNA-145	No effect on EWS::FLI1 expression	Augments EWS::FLI1 signature
FOXO1 (98, 175)	Downregulated by EWS::FLI1	Yes	No effect on EWS::FLI1 expression	De-represses genes downregulated by EWS::FLI1

NR, not reported.

Other transcription factors can instead counter-balance EWS::FLI1 activity. The developmental limb patterning TF HOXD13, for example, is upregulated by EWS::FLI1 but induces mesenchymal gene programs that are repressed by EWS::FLI1 (22). HOXD13 therefore partly antagonizes EWS::FLI1 function, and the competing activities of these TFs determine transcriptional cell states along a mesenchymal axis (22). Besides HOXD13, the other posterior HOXD proteins HOXD11 and HOXD10 have been identified as mediators of Ewing sarcoma oncogenic and metastatic phenotypes (86–88). Interestingly, in a recent study it was found that EWS::ETS-bound super-enhancers shared across 18 cell lines were enriched for *HOX* genes, suggesting a more global role for EWS::FLI1-mediated dysregulation of developmental HOX programs (89). Another critical TF highly expressed by Ewing sarcoma cells is ZEB2, which can induce expression of epithelial-mesenchymal transition (EMT) genes during normal development and in some cancers. ZEB2 positively regulates mesenchymal genes and migration and negatively regulates expression of epithelial genes in Ewing sarcoma cells (90). A follow-up study found that NKX2-2 target genes, which overlap significantly with EWS::FLI1 target genes, were inversely regulated by ZEB2 (25, 30, 38, 47). These data suggest that both HOXD13 and ZEB2 function to antagonize EWS::FLI1 function through activation/de-repression of EWS::FLI1-repressed genes.

The neurodevelopmental TF NKX2-2 is a target gene of EWS::FLI1 and it regulates a large subset of both the EWS::FLI1-activated and -repressed gene signature (30, 47). Indeed, NKX2-2 knockdown partially phenocopies the EWS::FLI1 “low” transcriptional state (25, 30, 38, 47). NR0B1, a TF that is involved in endocrine organ development, is also positively regulated by EWS::FLI1 and it contributes to EWS::FLI1-mediated gene repression (25, 30, 38, 47, 91). Both NR0B1 and NKX2-2 inhibit expression of a subset of fusion-repressed genes by direct promoter binding and recruitment of HDACs. In addition, the neurodevelopmental TF BCL11B represses subsets of EWS::FLI1-repressed genes through interacting with the NuRD complex (48, 92). In these examples, EWS::FLI1-activated TFs serve as transcriptional repressors and execute the fusion’s repressive functions by recruiting repressive HDAC-containing chromatin complexes.

A recent study found that 14% of all TFs are regulated by EWS::FLI1, resulting in cascading downstream effects on the cell transcriptome (81). In addition to the TFs mentioned above, EWS::FLI1 can modulate the expression of the Hedgehog signaling mediator GLI1 (83, 93), pluripotency TF SOX2 (38, 51), the neural crest and myogenic developmental TF PAX7 (94, 95), neurodevelopmental TF EGR2 (96, 97), the blood and bone developmental TF SOX6 (45), FOXO1 (98), and AP1 (99). The effect of these TFs on EWS::FLI1 activity has yet to be fully investigated but we anticipate that at least some will engage in positive or negative feedback mechanisms that influence EWS::FLI1 activity.

## Cell extrinsic regulators of EWS::FLI1 activity

The tumor microenvironment (TME) can have profound effects on inter- and intra- tumor heterogeneity. Tumor cells integrate and respond to complex layers of inputs from their TME including signals from immune and other non-tumor stromal cells, interactions with physically and biochemically complex protein and carbohydrate matrices, cytokines, and metabolic and oxygen gradients. These create a shifting landscape of pressures in space and time that affect cell phenotypes related to growth, immune evasion, treatment resistance, and metastasis (3, 100, 101). As tumors progress the TME undergoes dynamic restructuring *via* influx of inflammatory and immune cells, extracellular matrix (ECM) deposition and remodeling, necrosis, and hypoxia (102). Many of these changes are best understood in carcinomas, which typically arise from well-organized epithelial tissues that are surrounded by restrictive layers of basement membrane and adjacent non-tumor stroma (103). The TME of sarcomas is distinct, as they arise from mesenchyme-derived cells in diverse connective tissue locations and are not surrounded by basement membranes (104, 105).

Though still relatively understudied in Ewing sarcoma, the TME is critically important for Ewing sarcoma progression, treatment resistance, and metastasis (106). Over 80% of Ewing sarcomas arise in bone (7), a tissue with a physically and biochemically distinct and highly organized local microenvironment. Primary bone tumors are associated with a worse prognosis than extraskeletal Ewing sarcoma (107) and patients with bone metastases have worse outcomes than patients with lung-only metastases (10). Interestingly, Ewing sarcomas arise most commonly during adolescence and young adulthood, a period of extensive bone remodeling during which time total bone mass usually doubles (108). Together, these clinical observations support speculation that signals from the bone TME contribute to Ewing sarcoma progression. However, only recently has the field begun to identify specific signals in the bone that can alter EWS::FLI1 activity and tumor cell phenotypes (109).

### Bone TMEs can promote the EWS::FLI1 “low” state and create a “vicious cycle” of osteolysis

**Figure 3:**
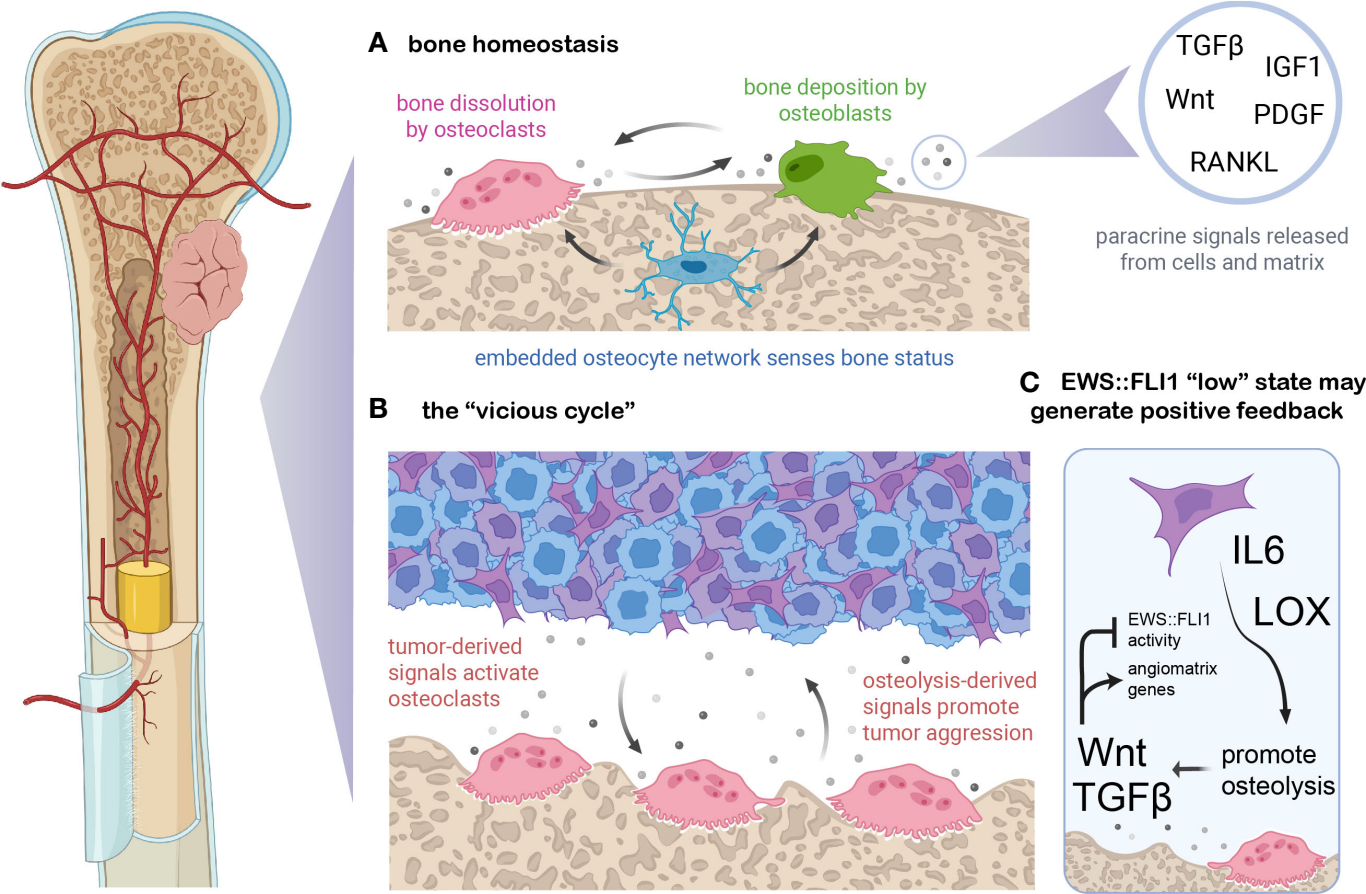
EWS::FLI1 activity is regulated by signals in the osteolytic bone tumor microenvironment. **(A)** Bone is constantly remodeled and homeostasis is maintained by osteoclasts (which resorb bone through protease and proton secretion), osteoblasts (which deposit mineralized, collagenous matrix), and osteoblast-derived osteocytes which form a network within bone and modulate osteoclast and osteoblast activity (110). Soluble signals exist either embedded in the bone matrix (and released upon dissolution) or generated *via* paracrine secretion from bone cells. These signals couple remodeling to deposition and shifts in these signals can either balance bone remodeling, favor deposition during periods of bone growth, or favor osteolysis during regression or calcium deficiency. **(B)** Ewing sarcoma cells can secrete numerous signals which dysregulate this balance, including osteoblast-activating factors such as RANKL, IL6, TNF-alpha, and LOX (109, 111–113). In turn, the dissolution of bone matrix releases embedded growth factors such Wnts, TGF-beta, PDGFs, FGFs, and IGF1 which can promote tumor growth and/or metastasis (111). **(C)** EWS::FLI1 “low” cells may generate a positive feedback loop with the osteolytic “vicious cycle”. Recent findings suggest that bone-derived signals such as Wnt and TGF-beta partially antagonize EWS::FLI1 activity, promoting an EWS::FLI1 “low” transcriptional state that upregulates mesenchymal-identify genes including pro-metastatic ECM molecules and angiogenesis-inducing genes (23). EWS::FLI1 antagonism also induces expression of LOX and IL-6, known promoters of osteolysis (17, 35, 114). Figure created with BioRender.com.

Bone is a highly dynamic tissue that undergoes constant remodeling to maintain a balance between bone density and blood calcium levels (110) (**Figure 3A**). Normal bone maintenance proceeds through the actions of several cell types. Mesenchymal stem cell-derived osteoblasts secrete type I collagens and other ECM proteins which combine with hydroxyapatite to form the structural basis of the biomineralized bone matrix (110, 115). Hematopoietic stem cell-derived osteoclasts break down bone *via* hydrogen ion and protease secretion, releasing calcium and soluble signals which feedback to osteoblasts, coupling bone remodeling and bone formation to maintain homeostasis (116). A network of bone-embedded osteocytes, derived from osteoblasts, integrates cues such as mechanical loading and hormonal signals and in turn secretes osteoblast and osteoclast modifying factors (117). Multiple feedback loops and reciprocal ligand-receptor interactions among these three cell types and others maintain a delicate balance of resorption and deposition in bone (111, 118). Several of the most critical signaling molecules that maintain this balance are RANKL, Wnt, TGF-beta, IGF, and PDGF ligands (116, 118, 119). As Ewing sarcomas frequently arise in and metastasize to this tissue, it is critical to understand the signals present in both normal and colonized or injured bone and how they may influence tumor cell states.

Soluble signals released by bone remodeling have profound effects on Ewing sarcoma phenotypes, promoting mesenchymal identity and invasive potential (109). This is partly due to the osteolytic “vicious cycle” which is characteristic of Ewing sarcoma and other primary and metastatic tumors that occur in bone (**Figure 3B**) (109, 111). Tumor cells can activate osteoclasts and bone resorption *via* secretion of proteins like RANKL, IL-6, LOX, and TNF-alpha (109, 111–113). Osteolysis stimulated by these signals then releases matrix-embedded growth factors and signals, which in turn promote tumor cell proliferation and invasion which can further promote osteolysis (109, 111). RANKL and IL-6 have been implicated in Ewing sarcoma paracrine signaling and metastatic progression (114, 120, 121). In addition, IL-6 and LOX are upregulated in EWS::FLI1 “low” cell states, suggesting that EWS::FLI1 “low” cells may have an enhanced propensity to initiate bone remodeling, though this remains to be rigorously tested (17, 35, 114) (**Figure 3C**).

Regardless of how bone remodeling is initiated, dissolution of the bone matrix will release a number of soluble factors that normally regulate osteoblast and osteoclast homeostasis, such as TGF-beta, IGF, and Wnt ligands, all of which can also alter tumor phenotypes (110, 122, 123). EWS::FLI1 expression plays an important role in regulating the sensitivity of Ewing sarcoma cells to these bone-derived signals. Notably, the TGF-beta receptor TGFBR2 is directly repressed by EWS::FLI1, inhibiting TGF-beta pathway activation, ECM gene expression/protein secretion, and pro-migratory signaling in EWS::FLI1 “high” cells (23, 35, 124). Activation of canonical Wnt/beta-catenin signaling in Ewing sarcoma cells results in derepression of TGFBR2 and creates an EWS::FLI1 “low” cell state without altering the level of the fusion protein (23, 124, 125). Another recent study found that inhibition of Wnt signaling using a Porcupine inhibitor reduced migration of Ewing sarcoma cells *in vitro* and spontaneous distant metastasis in an orthotopic pretibial xenograft model (126). Increases in Wnt activity lead to increased secretion of TGF-beta ligands as well as ECM proteins from tumor cells that contribute to an angiogenic and pro-tumorigenic TME (125). Thus, autocrine and paracrine feedback loops are created in the bone TME between transcriptionally heterogeneous tumor cells and the surrounding stroma, potentially accelerating the vicious cycle of osteolysis. Together these studies point to a key role for the bone TME in promoting metastatic progression by creating EWS::FLI1 “low”-like cell states. Targeted mechanistic studies in relevant model systems are now required to definitively test this intriguing hypothesis.

### EWS::FLI1 “low” states can directly remodel ECM and the TME

Changes to the ECM and ECM-related proteins have been shown to promote metastasis across many cancer types (100). Many genes that are repressed by EWS::FLI1 such as numerous collagens, laminin, SPARC, ECM1, fibrillin, tenascin-C, MGP, and fibronectin are important components of the ECM (100, 127, 128). EWS::FLI1 “low” cells have upregulated expression of these key ECM genes and also ECM-engaging proteins including integrins (e.g. ITGA1, ITGA4, and ITGB1), NCAM1, and dystroglycan (17, 127). These genes are normally repressed in EWS:FLI1 “high” cells, however in EWS::FLI1 “low” cells their expression is derepressed through unknown mechanisms. LOX, a matrix cross-linking protein implicated in fibrosis and metastasis (129, 130), is repressed by the fusion and has been identified as a marker of the EWS::FLI1 “low” cell state (17). Additionally, when Ewing sarcoma cell lines are exposed to Wnt3a and TGF-beta they acquire EWS::FLI1 “low” properties and increase expression and secretion of these and other ECM-related genes and proteins (23, 124, 125). Notably several of these proteins and proteoglycans, including collagens, SPARC, biglycan, and tenascin-C, are associated with angiogenesis and metastatic progression in many cancer types (100, 124, 131). The combination of Wnt and TGF-beta pathway activation is sometimes but not always required to fully activate this ECM program in Ewing sarcoma cells, again pointing to the critical contribution of spatially and temporally dynamic tumor:TME crosstalk in regulating EWS::FLI1-dependent transcriptional states, tumor cell heterogeneity, and phenotypic plasticity (124).

### Metabolic programs influence EWS::FLI1 activity

Large areas of normal bone are typically hypoxic, usually below 5% pO2 (113). Hypoxia regulates osteoclastogenesis and may contribute to the osteolytic vicious cycle in tumors (113). In addition, hypoxia is common in growing tumors that are metabolically active and have outstripped their blood supply. In carcinomas, these features are associated with increased EMT, immune evasion, and metastatic potential (132). There is growing evidence that hypoxia alters Ewing sarcoma cell state to support metastatic phenotypes, and this may involve modulation of EWS::FLI1 activity. One study found that exposure of Ewing sarcoma cells to hypoxia resulted in upregulation of EWS::FLI1-repressed targets and downregulation of EWS::FLI1-activated targets (133). Paradoxically however, hypoxia can lead to increased expression of EWS::FLI1 suggesting that low oxygen tension antagonizes transcriptional activity of the fusion indirectly (133, 134). More recently, it was shown in Ewing sarcoma models that hypoxia promotes osseous metastasis (135). Additional studies are now needed to understand the direct and indirect effects of hypoxia on EWS::FLI1 expression, activity, and cell state, and how these factors influence disease progression and metastatic dissemination.

Cancer cells commonly alter their metabolism to sustain the energetic and biosynthetic needs of uncontrolled proliferation and growth (4). In nearly a century since the Warburg hypothesis was published, the relationship between tumor aggression and enhanced glycolytic metabolism has been explored extensively (136) and increased glycolysis is known to contribute to the pro-metastatic effects of hypoxia (132, 137). Ewing sarcomas, like many other cancers, have high glycolytic metabolic rates relative to normal tissues and are sensitive to inhibitors of glycolysis like 2-DG (138). EWS::FLI1 is a master regulator of metabolic reprogramming in Ewing sarcoma cells and EWS::FLI1 knockdown increases glycolytic output (139). In addition, EWS::FLI1 activates high activity of serine glycine one carbon (SGOC) metabolism, rendering the cells sensitive to SGOC pathway inhibition (139–142). EWS::FLI1 reduction also results in accumulation of reactive oxygen species, and dysregulation of other major metabolic pathways (139, 140). Finally, recent single cell analyses of Ewing sarcoma PDXs linked EWS::FLI1 activity and metabolic state, revealing that differential transcriptional signatures of glycolysis and hypoxia are positively correlated with the level of fusion activity (33). The plasticity of metabolic states and how they align with or influence EWS::FLI1 “high” and EWS::FLI1 “low” cell states will need to be more deeply explored, especially as metabolic therapies move forward into clinical trials.

## Therapeutic implications of EWS::FLI1 “high” and “low” cell states

In the prior sections we have described the existence and phenotypic characteristics of EWS::FLI1 “high” and “low” cell populations in Ewing sarcoma and broadly summarized what is currently known about the complex network of cell intrinsic and extrinsic factors that modulate these states. In the following section we will review the potential therapeutic implications of these distinct cell states, both in terms of response to current therapies and as considerations for novel targeted approaches including immunotherapy.

### Cytotoxic agents and small molecules can inhibit EWS::FLI1 activity

Given the exquisite dependency of Ewing sarcoma on EWS::ETS driver fusions, pharmacologic approaches to inhibit their expression or activity have long been sought and several candidate drugs have been identified (**Table 2**). These drugs include mithramycin (143), cytarabine (ARA-C) (144), doxorubicin (144), trabectedin (145), and rapamycin (146) as well as the investigational agent YK-4-279 (147). Mithramycin and YK-4-279 do not alter the level of fusion expression but disrupt its transcriptional activity (143, 147). ARA-C and doxorubicin both induce loss of EWS::FLI1 protein expression and this partially reverses the EWS::FLI1 gene signature (144). Likewise, the mTOR inhibitor rapamycin has also been reported to lead to reduction of EWS::FLI1 protein expression (146). The alkylating agent trabectedin is a strong modulator of EWS::FLI1 activity and induces apoptosis in Ewing sarcoma cells (145, 148). Though the preclinical studies for all of these agents have been highly promising, and their effects on inhibiting the EWS::FLI1 gene signature are profound, clinical results have thus far been disappointing. While complex and diverse reasons are likely to underlie this, consideration must be given to the possibility that creation of subpopulations of EWS::FLI1 “low” cells by these treatments might actually support tumor progression.

**Table 2:** Summary of selected drugs and small molecules and their effects on EWS::FLI1 and its gene signatures.

Treatment	Drug target or function	Effect on EWS::FLI1 expression	Effect on EWS::FLI1 targets
Mithramycin (143)	Binds GC rich regions	Does not alter expression	Disrupts signature, reduces activated targets
ARA-C (144)	Nucleoside analog	Reduces EWS::FLI1 protein expression	Disrupts signature, partly mimics EWS::FLI1 knockdown
Doxorubicin (144)	Standard of care chemotherapy, topoisomerase II poison	Reduces EWS::FLI1 protein expression	Disrupts signature, partly mimics EWS::FLI1 knockdown
Trabectidin (148)	DNA binding, transcriptional interference	Does not alter expression	Disrupts signature
YK-4-279 (147, 171)	Interrupts RNA helicase A binding	Does not alter expression	Disrupts signature
Rapamycin (146)	mTOR inhibitor	Reduces EWS::FLI1 expression	Disrupts signature
HDACi (multiple drugs) (37, 58, 155, 156)	HDAC inhibitors	Yes or no, depending on drug and dose	Can disrupt or reverse signature
JQ1 (73, 151)	Bromodomain inhibitor	Reduces EWS::FLI1 expression	Can disrupt or reverse signature, partly mimics EWS::FLI1 knockdown
HCI2509 (49, 72)	LSD1 inhibitor	NR	Can disrupt or reverse signature, partly mimics EWS::FLI1 knockdown
JIB-04	Histone demethylase inhibitor	Increases EWS::FLI1 expression	Can disrupt or reverse signature, partly mimics EWS::FLI1 knockdown

Due to the reliance of EWS::FLI1 on chromatin remodeling complexes, there is great interest in targeting Ewing sarcoma and the fusion specifically using epigenetic drugs including agents that inhibit HDACs (HDACi) (149), bromodomain proteins (BETi) (73, 150–152), LSD1 (LSD1i) (49, 72), and KDM3A (JIB-04) (153). Use of HDACi *in vitro* and *in vivo* inhibits Ewing sarcoma viability, proliferation, and tumor growth (154–156). HDACi can directly alter expression of the fusion protein (58, 155, 156) and indirectly affect transcriptional function by reactivating expression of repressed target genes (37, 154, 155). BET inhibitors induce cell cycle arrest and partially reverse expression of the EWS::FLI1 gene signature (73, 150–152). Likewise, LSD1 inhibitors have similar effects on cell phenotype and EWS::FLI1 transcriptional activity (Reviewed in (72)). Very promising preclinical data with HDAC, BET and LSD1 inhibitors have led to inclusion of pediatric Ewing sarcoma patients in early phase clinical trials that are ongoing with these agents (NCT02909777, NCT03600649; NCT03936465). Overall, investigation of epigenetic drugs for Ewing sarcoma treatment is still in its infancy but there is clear evidence that these agents can disrupt EWS::FLI1 transcriptional activity and inhibit fusion-driven gene signatures. As with cytotoxic drugs, it will be important to establish how these disruptions impact on cell state and whether Ewing sarcoma tumor cells that are subject to epigenetic modification adopt more mesenchymal, and possibly more metastatic, properties.

### EWS::FLI1 “high” and “low” states and the immune microenvironment

Like most pediatric solid tumors Ewing sarcoma generates relatively few neoantigens that can stimulate an antigen-specific immune response (157). Ewing sarcomas have low immune cell infiltration in general compared to other tumors and other sarcomas, and while an increase in infiltration has been observed in some relapsed *vs.* primary tumors (158) overall T-cell infiltration does not consistently correlate with better prognosis (159, 160). Ewing sarcoma tumors also frequently downregulate HLA class I expression, which may help tumor cells evade certain types of immune targeting (161). In keeping with these observations, CAR-T based therapies, which have been revolutionary in hematological malignancies, have thus far largely failed to have an impact in Ewing sarcoma or other pediatric solid tumors (162).

Despite these roadblocks, recent studies suggest that immune therapies are worthy of exploration in Ewing sarcoma, especially when considering tumor heterogeneity and EWS::FLI1 “high” and “low” states. EWS::FLI1 “low” cells are more sensitive to T-cell mediated killing than EWS::FLI1 “high” cells, possibly due to differences in expression of adhesion molecules as well as upregulated expression of PD-L1 and PD-L2 which renders them more sensitive to immune checkpoint inhibition (163). EWS::FLI1 knockdown leads to upregulation of IL-6 and other cytokines such as CXCL1, CCL3, and GM-CSF that are immunomodulatory (114). Immunosuppressive myeloid derived suppressor cells (MDSCs) have also been identified in Ewing sarcoma patients (164) and may play a role in tumor progression, particularly in the context of EWS::FLI1 “low” cell states. Genes normally repressed by EWS::FLI1 but upregulated in EWS::FLI1 "low states", including LOX, TGF-beta, FN1, SPARC, and NT5E, have been implicated in MDSC recruitment or activity in other cancers (165–167).

These hypotheses remain speculative, but EWS::FLI1-dependent heterogeneity in immune suppressive and immune activating gene expression may represent an important dimension of how the TME and Ewing sarcoma reciprocally interact. However, in the absence of immunocompetent preclinical animal models it has been challenging to elucidate how these signals affect tumor outgrowth and metastasis. The creation of humanized mouse models (168) and an immune competent zebrafish model (169) of Ewing sarcoma should enable new studies that can dissect the reciprocal interactions between EWS::FLI1 activity, tumor cell heterogeneity, and the immune TME and how they might impact on immunotherapy.

## Future outlook for our understanding of EWS::FLI1 regulation and cell state heterogeneity

The field is still establishing a consensus regarding how EWS::FLI1 “high” and “low” cells differ transcriptionally and phenotypically. But, broadly, high EWS::FLI1 activity appears to contribute to proliferation and transformation, while EWS::FLI1 antagonism permits re-expression of mesenchymal and metastasis-associated gene programs. Here we have highlighted many cell-intrinsic factors which alter EWS::FLI1 activity and expression, including gene programs that regulate *EWS::FLI1* mRNA generation and stability, proteasomal degradation that keeps EWS::FLI1 levels below a toxic threshold, and partnerships with other TFs and chromatin remodeling complexes that serve to moderate fusion-dependent gene activation and/or gene repression. These findings highlight that EWS::FLI1 cannot act alone and that it relies on a myriad of other components to tightly regulate its expression and transcriptional activity. We also reviewed what is currently known about non-cell-autonomous factors that modulate EWS::FLI1 “high” and “low” phenotypes. This includes antagonism of EWS::FLI1 gene repression by signals present in the bone TME (in particular Wnt and TGF-beta ligands), the critical contribution of ECM proteins, possible differential interactions with the immune TME, and EWS::FLI1 activity-targeting drug treatments. Overlapping combinations of these intrinsic and extrinsic factors turn a genetically “simple” tumor into a highly heterogeneous mixture of multiple distinct cell states and phenotypes. This raises obvious challenges for clinical treatment of Ewing sarcoma, but also suggests that molecular interrogation of this cell state axis may reveal targetable vulnerabilities.

Improvements in outcomes for patients with relapsed or metastatic Ewing sarcoma will require novel and more biologically targeted agents (170). The studies highlighted in this review provide an important foundation for our understanding of EWS::FLI1-dependent, heterogeneous cell states and the many biological factors that regulate them. Recent findings that EWS::FLI1 “low” cell states are important for metastasis creates new challenges and opportunities for therapeutic advances. Treatments effective against EWS::FLI1 “high” cells states may not effectively target EWS::FLI1 “low” cells, which are transcriptionally and phenotypically distinct. In addition, drugs designed to inhibit EWS::FLI1 activity that do not completely eradicate tumors could generate a residual population of more aggressive EWS::FLI1 “low” cells. The plastic nature of these cell states may allow EWS::FLI1 “low” cells that persist through treatment to re-establish heterogeneous tumors with both EWS::FLI1 “high” and “low” cells, though this remains to determined. Future treatment strategies may involve combinatorial approaches that successfully target cells across a range of EWS::FLI1 activities, preventing the survival of more resistant and aggressive subpopulations. Alternatively, drugs that target the molecular underpinnings of epigenetic plasticity may “trap” Ewing sarcoma cells in a narrower range of cell states, limiting intratumoral heterogeneity and increasing treatment effectiveness. These yet untested hypotheses should be high priorities for future investigation.

## Author contributions

All authors contributed to conception, writing, and editing of the final manuscript. AA and EW contributed equally to literature review and first draft. All authors contributed to the article and approved the submitted version.

## Funding

The authors acknowledge grant support from: NIH/NCI R01 CA215981 (ERL), F31CA247104 (AA); AACR-QuadW Sarcoma Fellowship in Memory of Willie Tichenor (EDW); and the 1M4Anna Foundation (ERL).

## Acknowledgments

The authors wish to thank their many colleagues in the international Ewing sarcoma research community for their tireless efforts to advance knowledge that will lead to improved outcomes for patients. We apologize that many important citations have not been included due to space limitations.

## Conflict of interest

The authors declare that the research was conducted in the absence of any commercial or financial relationships that could be construed as a potential conflict of interest.

## Publisher’s note

All claims expressed in this article are solely those of the authors and do not necessarily represent those of their affiliated organizations, or those of the publisher, the editors and the reviewers. Any product that may be evaluated in this article, or claim that may be made by its manufacturer, is not guaranteed or endorsed by the publisher.

## References

[1] Greaves M. Evolutionary determinants of cancer. Cancer Discov (2015) 5(8):806–20. doi: 10.1158/2159-8290 PMC4539576 26193902

[2] McGranahan N Swanton C. Clonal heterogeneity and tumor evolution: Past, present, and the future. Cell (2017) 168(4):613–28. doi: 10.1016/j.cell.2017.01.018 28187284

[3] Li Z Seehawer M Polyak K. Untangling the web of intratumour heterogeneity. Nat Cell Biol (2022) 24(8):1192–201. doi: 10.1038/s41556-022-00969-x 35941364

[4] Hanahan D. Hallmarks of cancer: New dimensions. Cancer Discov (2022) 12(1):31–46. doi: 10.1158/2159-8290.cd-21-1059 35022204

[5] Sweet-Cordero EA Biegel JA. The genomic landscape of pediatric cancers: Implications for diagnosis and treatment. Science (2019) 363(6432):1170–5. doi: 10.1126/science.aaw3535 PMC7757338 30872516

[6] Filbin M Monje M. Developmental origins and emerging therapeutic opportunities for childhood cancer. Nat Med (2019) 25(3):367–76. doi: 10.1038/s41591-019-0383-9 PMC6631320 30842674

[7] Riggi N Suvà ML Stamenkovic I. Ewing's sarcoma. New Engl J Med (2021) 384(2):154–64. doi: 10.1056/NEJMra2028910 33497548

[8] Grünewald TGP Cidre-Aranaz F Surdez D Tomazou EM de Álava E Kovar H. Ewing Sarcoma. Nat Rev Dis Primers (2018) 4(1):5. doi: 10.1038/s41572-018-0003-x 29977059

[9] Ginsberg JP Goodman P Leisenring W Ness KK Meyers PA Wolden SL. Long-term survivors of childhood Ewing sarcoma: Report from the childhood cancer survivor study. J Natl Cancer Instit (2010) 102(16):1272–83. doi: 10.1093/jnci/djq278 PMC2948841 20656964

[10] Gaspar N Hawkins DS Dirksen U Lewis IJ Ferrari S Le Deley MC. Ewing Sarcoma: Current management and future approaches through collaboration. J Clin Oncol (2015) 33(27):3036–46. doi: 10.1200/jco.2014.59.5256 26304893

[11] Delattre O Zucman J Plougastel B Desmaze C Melot T Peter M. Gene fusion with an ets DNA-binding domain caused by chromosome translocation in human tumours. Nature (1992) 359(6391):162–5. doi: 10.1038/359162a0 1522903

[12] Sankar S Lessnick SL. Promiscuous partnerships in ewing's sarcoma. Cancer Genet (2011) 204(7):351–65. doi: 10.1016/j.cancergen.2011.07.008 PMC3164520 21872822

[13] Crompton BD Stewart C Taylor-Weiner A Alexe G Kurek KC Calicchio ML. The genomic landscape of pediatric Ewing sarcoma. Cancer Discovery (2014) 4(11):1326–41. doi: 10.1158/2159-8290.Cd-13-1037 25186949

[14] Brohl AS Solomon DA Chang W Wang J Song Y Sindiri S. The genomic landscape of the Ewing sarcoma family of tumors reveals recurrent Stag2 mutation. PloS Genet (2014) 10(7):e1004475. doi: 10.1371/journal.pgen.1004475 25010205 PMC4091782

[15] Anderson ND de Borja R Young MD Fuligni F Rosic A Roberts ND. Rearrangement bursts generate canonical gene fusions in bone and soft tissue tumors. Science (2018) 361(6405):eaam8419. doi: 10.1126/science.aam8419 30166462 PMC6176908

[16] Easwaran H Tsai HC Baylin SB. Cancer epigenetics: Tumor heterogeneity, plasticity of stem-like states, and drug resistance. Mol Cell (2014) 54(5):716–27. doi: 10.1016/j.molcel.2014.05.015 PMC4103691 24905005

[17] Franzetti GA Laud-Duval K van der Ent W Brisac A Irondelle M Aubert S. Cell-to-Cell heterogeneity of Ewsr1-Fli1 activity determines Proliferation/Migration choices in Ewing sarcoma cells. Oncogene (2017) 36(25):3505–14. doi: 10.1038/onc.2016.498 PMC5541267 28135250

[18] Chaturvedi A Hoffman LM Welm AL Lessnick SL Beckerle MC. The Ews/Fli oncogene drives changes in cellular morphology, adhesion, and migration in Ewing sarcoma. Genes Cancer (2012) 3(2):102–16. doi: 10.1177/1947601912457024 PMC3463921 23050043

[19] Seong BKA Dharia NV Lin S Donovan KA Chong S Robichaud A. Trim8 modulates the Ews/Fli oncoprotein to promote survival in Ewing sarcoma. Cancer Cell (2021) 39(9):1262–78.e7. doi: 10.1016/j.ccell.2021.07.003 34329586 PMC8443273

[20] Adane B Alexe G Seong BKA Lu D Hwang EE Hnisz D. Stag2 loss rewires oncogenic and developmental programs to promote metastasis in Ewing sarcoma. Cancer Cell (2021) 39(6):827–44.e10. doi: 10.1016/j.ccell.2021.05.007 34129824 PMC8378827

[21] Surdez D Zaidi S Grossetête S Laud-Duval K Ferre AS Mous L. Stag2 mutations alter ctcf-anchored loop extrusion, reduce cis-regulatory interactions and Ewsr1-Fli1 activity in Ewing sarcoma. Cancer Cell (2021) 39(6):810–26.e9. doi: 10.1016/j.ccell.2021.04.001 33930311

[22] Apfelbaum AA Wu F Hawkins AG Magnuson B Jimenez JA Taylor SD. Ews-Fli1 and Hoxd13 control tumor cell plasticity in Ewing sarcoma. Clin Cancer Res (2022) 28(20):4466–78. doi: 10.1158/1078-0432.Ccr-22-0384 PMC9588607 35653119

[23] Pedersen EA Menon R Bailey KM Thomas DG Van Noord RA Tran J. Activation of Wnt/β-catenin in Ewing sarcoma cells antagonizes Ews/Ets function and promotes phenotypic transition to more metastatic cell states. Cancer Res (2016) 76(17):5040–53. doi: 10.1158/0008-5472.can-15-3422 PMC5010452 27364557

[24] Bierbaumer L Katschnig AM Radic-Sarikas B Kauer MO Petro JA Högler S. Yap/Taz inhibition reduces metastatic potential of Ewing sarcoma cells. Oncogenesis (2021) 10(1):2. doi: 10.1038/s41389-020-00294-8 33419969 PMC7794350

[25] Kinsey M Smith R Lessnick SL. Nr0b1 is required for the oncogenic phenotype mediated by Ews/Fli in ewing's sarcoma. Mol Cancer Res MCR (2006) 4(11):851–9. doi: 10.1158/1541-7786.mcr-06-0090 17114343

[26] Deneen B Denny CT. Loss of P16 pathways stabilizes Ews/Fli1 expression and complements Ews/Fli1 mediated transformation. Oncogene (2001) 20(46):6731–41. doi: 10.1038/sj.onc.1204875 11709708

[27] Lessnick SL Dacwag CS Golub TR. The ewing's sarcoma oncoprotein Ews/Fli induces a P53-dependent growth arrest in primary human fibroblasts. Cancer Cell (2002) 1(4):393–401. doi: 10.1016/s1535-6108(02)00056-9 12086853

[28] Sohn EJ Li H Reidy K Beers LF Christensen BL Lee SB. Ews/Fli1 oncogene activates caspase 3 transcription and triggers apoptosis in Vivoews/Fli1 activates caspase 3. Cancer Res (2010) 70(3):1154–63. doi: 10.1158/0008-5472.CAN-09-1993 PMC2818579 20103643

[29] Chaturvedi A Hoffman LM Jensen CC Lin YC Grossmann AH Randall RL. Molecular dissection of the mechanism by which Ews/Fli expression compromises actin cytoskeletal integrity and cell adhesion in Ewing sarcoma. Mol Biol Cell (2014) 25(18):2695–709. doi: 10.1091/mbc.E14-01-0007 PMC4161506 25057021

[30] Fadul J Bell R Hoffman LM Beckerle MC Engel ME Lessnick SL. Ews/Fli utilizes Nkx2-2 to repress mesenchymal features of Ewing sarcoma. Genes Cancer (2015) 6(3-4):129–43. doi: 10.18632/genesandcancer.57 PMC4426950 26000096

[31] Segal D Mazloom-Farsibaf H Chang B-J Roudot P Rajendran D Daetwyler S. *In vivo* 3d profiling of site-specific human cancer cell morphotypes in zebrafish. J Cell Biol (2022) 221(11):e202109100. doi: 10.1083/jcb.202109100 36155740 PMC9516844

[32] Katschnig AM Kauer MO Schwentner R Tomazou EM Mutz CN Linder M. Ews-Fli1 perturbs Mrtfb/Yap-1/Tead target gene regulation inhibiting cytoskeletal autoregulatory feedback in Ewing sarcoma. Oncogene (2017) 36(43):5995–6005. doi: 10.1038/onc.2017.202 28671673 PMC5666320

[33] Aynaud M-M Mirabeau O Gruel N Grossetête S Boeva V Durand S. Transcriptional programs define intratumoral heterogeneity of Ewing sarcoma at single-cell resolution. Cell Rep (2020) 30(6):1767–79.e6. doi: 10.1016/j.celrep.2020.01.049 32049009

[34] Khoogar R Li F Chen Y Ignatius M Lawlor ER Kitagawa K. Single-cell rna profiling identifies diverse cellular responses to Ewsr1/Fli1 downregulation in Ewing sarcoma cells. Cell Oncol (Dordr) (2022) 45(1):19–40. doi: 10.1007/s13402-021-00640-x 34997546 PMC10959445

[35] Sankar S Bell R Stephens B Zhuo R Sharma S Bearss DJ. Mechanism and relevance of Ews/Fli-mediated transcriptional repression in Ewing sarcoma. Oncogene (2013) 32(42):5089–100. doi: 10.1038/onc.2012.525 PMC3899696 23178492

[36] Riggi N Knoechel B Gillespie SM Rheinbay E Boulay G Suvà ML. Ews-Fli1 utilizes divergent chromatin remodeling mechanisms to directly activate or repress enhancer elements in Ewing sarcoma. Cancer Cell (2014) 26(5):668–81. doi: 10.1016/j.ccell.2014.10.004 PMC4492343 25453903

[37] Tomazou EM Sheffield NC Schmidl C Schuster M Schönegger A Datlinger P. Epigenome mapping reveals distinct modes of gene regulation and widespread enhancer reprogramming by the oncogenic fusion protein ews-Fli1. Cell Rep (2015) 10(7):1082–95. doi: 10.1016/j.celrep.2015.01.042 PMC4542316 25704812

[38] Boulay G Volorio A Iyer S Broye LC Stamenkovic I Riggi N. Epigenome editing of microsatellite repeats defines tumor-specific enhancer functions and dependencies. Genes Dev (2018) 32(15-16):1008–19. doi: 10.1101/gad.315192.118 PMC6075149 30042132

[39] Minas TZ Surdez D Javaheri T Tanaka M Howarth M Kang HJ. Combined experience of six independent laboratories attempting to create an Ewing sarcoma mouse model. Oncotarget (2017) 8(21):34141–63. doi: 10.18632/oncotarget.9388 PMC5470957 27191748

[40] Cidre-Aranaz F Alonso J. Ews/Fli1 target genes and therapeutic opportunities in Ewing sarcoma. Front Oncol (2015) 5:162. doi: 10.3389/fonc.2015.00162 26258070 PMC4507460

[41] Kauer M Ban J Kofler R Walker B Davis S Meltzer P. A molecular function map of ewing's sarcoma. PloS One (2009) 4(4):e5415. doi: 10.1371/journal.pone.0005415 19404404 PMC2671847

[42] Postel-Vinay S Véron AS Tirode F Pierron G Reynaud S Kovar H. Common variants near tardbp and Egr2 are associated with susceptibility to Ewing sarcoma. Nat Genet (2012) 44(3):323–7. doi: 10.1038/ng.1085 22327514

[43] Machiela MJ Grünewald TGP Surdez D Reynaud S Mirabeau O Karlins E. Genome-wide association study identifies multiple new loci associated with Ewing sarcoma susceptibility. Nat Commun (2018) 9(1):3184. doi: 10.1038/s41467-018-05537-2 30093639 PMC6085378

[44] Monument MJ Johnson KM Grossmann AH Schiffman JD Randall RL Lessnick SL. Microsatellites with macro-influence in Ewing sarcoma. Genes (2012) 3(3):444–60. doi: 10.3390/genes3030444 PMC3899989 24704979

[45] Marchetto A Ohmura S Orth MF Knott MML Colombo MV Arrigoni C. Oncogenic hijacking of a developmental transcription factor evokes vulnerability toward oxidative stress in Ewing sarcoma. Nat Commun (2020) 11(1):2423. doi: 10.1038/s41467-020-16244-2 32415069 PMC7228971

[46] Theisen ER Selich-Anderson J Miller KR Tanner JM Taslim C Pishas KI. Chromatin profiling reveals relocalization of lysine-specific demethylase 1 by an oncogenic fusion protein. Epigenetics (2021) 16(4):405–24. doi: 10.1080/15592294.2020.1805678 PMC7993145 32842875

[47] Owen LA Kowalewski AA Lessnick SL. Ews/Fli mediates transcriptional repression*Via* Nkx2.2 during oncogenic transformation in ewing's sarcoma. PloS One (2008) 3(4):e1965. doi: 10.1371/journal.pone.0001965 18414662 PMC2291578

[48] Marques Howarth M Simpson D Ngok SP Nieves B Chen R Siprashvili Z. Long noncoding rna Ewsat1-mediated gene repression facilitates Ewing sarcoma oncogenesis. J Clin Invest (2014) 124(12):5275–90. doi: 10.1172/JCI72124 PMC4348951 25401475

[49] Sankar S Theisen ER Bearss J Mulvihill T Hoffman LM Sorna V. Reversible Lsd1 inhibition interferes with global Ews/Ets transcriptional activity and impedes Ewing sarcoma tumor growth. Clin Cancer Res (2014) 20(17):4584–97. doi: 10.1158/1078-0432.Ccr-14-0072 PMC4155010 24963049

[50] Tirode F Laud-Duval K Prieur A Delorme B Charbord P Delattre O. Mesenchymal stem cell features of Ewing tumors. Cancer Cell (2007) 11(5):421–9. doi: 10.1016/j.ccr.2007.02.027 17482132

[51] Riggi N Suvà ML De Vito C Provero P Stehle JC Baumer K. Ews-Fli-1 modulates Mirna145 and Sox2 expression to initiate mesenchymal stem cell reprogramming toward Ewing sarcoma cancer stem cells. Genes Dev (2010) 24(9):916–32. doi: 10.1101/gad.1899710 PMC2861191 20382729

[52] Riggi N Suvà ML Suvà D Cironi L Provero P Tercier S. Ews-Fli-1 expression triggers a ewing's sarcoma initiation program in primary human mesenchymal stem cells. Cancer Res (2008) 68(7):2176–85. doi: 10.1158/0008-5472.can-07-1761 18381423

[53] Sole A Grossetête S Heintzé M Babin L Zaïdi S Revy P. Unraveling Ewing sarcoma tumorigenesis originating from patient-derived mesenchymal stem cells. Cancer Res (2021) 81(19):4994–5006. doi: 10.1158/0008-5472.Can-20-3837 34341072 PMC8487988

[54] von Levetzow C Jiang X Gwye Y von Levetzow G Hung L Cooper A. Modeling initiation of Ewing sarcoma in human neural crest cells. PloS One (2011) 6(4):e19305. doi: 10.1371/journal.pone.0019305 21559395 PMC3084816

[55] Lin PP Wang Y Lozano G. Mesenchymal stem cells and the origin of ewing's sarcoma. Sarcoma (2011) 2011:276463. doi: 10.1155/2011/276463 20953407 PMC2952797

[56] Miller HE Gorthi A Bassani N Lawrence LA Iskra BS Bishop AJR. Reconstruction of Ewing sarcoma developmental context from mass-scale transcriptomics reveals characteristics of Ewsr1-Fli1 permissibility. Cancers (2020) 12(4):948. doi: 10.3390/cancers12040948 32290418 PMC7226175

[57] Sheffield NC Pierron G Klughammer J Datlinger P Schönegger A Schuster M. DNA Methylation heterogeneity defines a disease spectrum in Ewing sarcoma. Nat Med (2017) 23(3):386–95. doi: 10.1038/nm.4273 PMC5951283 28134926

[58] García-Domínguez DJ Hajji N Sánchez-Molina S Figuerola-Bou E de Pablos RM Espinosa-Oliva AM. Selective inhibition of Hdac6 regulates expression of the oncogenic driver Ewsr1-Fli1 through the Ewsr1 promoter in Ewing sarcoma. Oncogene (2021) 40(39):5843–53. doi: 10.1038/s41388-021-01974-4 PMC8484017 34345016

[59] Grohar Patrick J Kim S Rangel Rivera Guillermo O Sen N Haddock S Harlow Matt L. Functional genomic screening reveals splicing of the ews-Fli1 fusion transcript as a vulnerability in Ewing sarcoma. Cell Rep (2016) 14(3):598–610. doi: 10.1016/j.celrep.2015.12.063 26776507 PMC4755295

[60] Keskin T Bakaric A Waszyk P Boulay G Torsello M Cornaz-Buros S. Lin28b underlies the pathogenesis of a subclass of Ewing sarcoma. Cell Rep (2020) 30(13):4567–83.e5. doi: 10.1016/j.celrep.2020.107539 32234488

[61] Barrett C Budhiraja A Parashar V Batish M. The landscape of regulatory noncoding rnas in ewing’s sarcoma. Biomedicines (2021) 9(8):933. doi: 10.3390/biomedicines9080933 34440137 PMC8391329

[62] Aryee DNT Fock V Kapoor U Radic-Sarikas B Kovar H. Zooming in on long non-coding rnas in Ewing sarcoma pathogenesis. Cells (2022) 11(8):1267. doi: 10.3390/cells11081267 35455947 PMC9032025

[63] Dylla L Moore C Jedlicka P. Micrornas in Ewing sarcoma. Front Oncol (2013) 3:65. doi: 10.3389/fonc.2013.00065 23543617 PMC3610014

[64] Ban J Jug G Mestdagh P Schwentner R Kauer M Aryee DN. Hsa-Mir-145 is the top ews-Fli1-Repressed microrna involved in a positive feedback loop in ewing's sarcoma. Oncogene (2011) 30(18):2173–80. doi: 10.1038/onc.2010.581 PMC4959567 21217773

[65] McKinsey EL Parrish JK Irwin AE Niemeyer BF Kern HB Birks DK. A novel oncogenic mechanism in Ewing sarcoma involving igf pathway targeting by Ews/Fli1-regulated micrornas. Oncogene (2011) 30(49):4910–20. doi: 10.1038/onc.2011.197 PMC4696862 21643012

[66] De Vito C Riggi N Suva M-L Janiszewska M Horlbeck J Baumer K. Let-7a is a direct ews-Fli-1 target implicated in ewing's sarcoma development. PloS One (2011) 6(8):e23592. doi: 10.1371/journal.pone.0023592 21853155 PMC3154507

[67] Stolte B Iniguez AB Dharia NV Robichaud AL Conway AS Morgan AM. Genome-scale crispr-Cas9 screen identifies druggable dependencies in Tp53 wild-type Ewing sarcoma. J Exp Med (2018) 215(8):2137–55. doi: 10.1084/jem.20171066 PMC6080915 30045945

[68] Gierisch ME Pedot G Walser F Lopez-Garcia LA Jaaks P Niggli FK. Usp19 deubiquitinates ews-Fli1 to regulate Ewing sarcoma growth. Sci Rep (2019) 9(1):1–12. doi: 10.1038/s41598-018-37264-5 30700749 PMC6353870

[69] Su S Chen J Jiang Y Wang Y Vital T Zhang J. Spop and Otud7a control ews–Fli1 protein stability to govern Ewing sarcoma growth. Adv Sci (2021) 8(14):2004846. doi: 10.1002/advs.202004846 PMC8292909 34060252

[70] Hahm K Cho K Lee C Im Y Choi S Sorensen P. The ews-Fli1 oncogene of Ewing sarcoma represses tgf-s type ii receptor gene expression. Nat Genet (1999) 23:222–7. doi: 10.1038/13854 10508522

[71] Prieur A Tirode F Cohen P Delattre O. Ews/Fli-1 silencing and gene profiling of Ewing cells reveal downstream oncogenic pathways and a crucial role for repression of insulin-like growth factor binding protein 3. Mol Cell Biol (2004) 24(16):7275–83. doi: 10.1128/MCB.24.16.7275-7283.2004 PMC479730 15282325

[72] Theisen ER Pishas KI Saund RS Lessnick SL. Therapeutic opportunities in Ewing sarcoma: Ews-fli inhibition *Via* Lsd1 targeting. Oncotarget (2016) 7(14):17616. doi: 10.18632/oncotarget.7124 26848860 PMC4951237

[73] Gollavilli PN Pawar A Wilder-Romans K Natesan R Engelke CG Dommeti VL. Ews/Ets-driven Ewing sarcoma requires bet bromodomain proteinsidentification of Phf19 as an essential Ews/Ets target. Cancer Res (2018) 78(16):4760–73. doi: 10.1158/0008-5472.CAN-18-0484 29898995

[74] Gierisch ME Pfistner F Lopez-Garcia LA Harder L Schäfer BW Niggli FK. Proteasomal degradation of the ews-Fli1 fusion protein is regulated by a single lysine residue. J Biol Chem (2016) 291(52):26922–33. doi: 10.1074/jbc.M116.752063 PMC5207197 27875302

[75] Dharia NV Kugener G Guenther LM Malone CF Durbin AD Hong AL. A first-generation pediatric cancer dependency map. Nat Genet (2021) 53(4):529–38. doi: 10.1038/s41588-021-00819-w PMC8049517 33753930

[76] Tirode F Surdez D Ma X Parker M Le Deley MC Bahrami A. Genomic landscape of Ewing sarcoma defines an aggressive subtype with Co-association of Stag2 and Tp53 mutations. Cancer Discov (2014) 4(11):1342–53. doi: 10.1158/2159-8290.Cd-14-0622 PMC4264969 25223734

[77] Parrish J Sechler M Winn R Jedlicka P. The histone demethylase Kdm3a is a microrna-22-Regulated tumor promoter in Ewing sarcoma. Oncogene (2015) 34(2):257–62. doi: 10.1038/onc.2013.541 PMC4477825 24362521

[78] Sechler M Parrish JK Birks DK Jedlicka P. The histone demethylase Kdm3a, and its downstream target mcam, promote Ewing sarcoma cell migration and metastasis. Oncogene (2017) 36(29):4150–60. doi: 10.1038/onc.2017.44 PMC5519422 28319067

[79] Lin L Huang M Shi X Mayakonda A Hu K Jiang YY. Super-Enhancer-Associated Meis1 promotes transcriptional dysregulation in Ewing sarcoma in Co-operation with ews-Fli1. Nucleic Acids Res (2019) 47(3):1255–67. doi: 10.1093/nar/gky1207 PMC6379679 30496486

[80] Bledsoe KL McGee-Lawrence ME Camilleri ET Wang X Riester SM van Wijnen AJ. Runx3 facilitates growth of Ewing sarcoma cells. J Cell Physiol (2014) 229(12):2049–56. doi: 10.1002/jcp.24663 PMC4149590 24812032

[81] García-García L Fernández-Tabanera E Cervera ST Melero-Fernández de Mera RM Josa S González-González L. The transcription factor Fezf1, a direct target of Ewsr1-Fli1 in Ewing sarcoma cells, regulates the expression of neural-specific genes. Cancers (2021) 13(22):5668. doi: 10.3390/cancers13225668 34830820 PMC8616448

[82] Bilke S Schwentner R Yang F Kauer M Jug G Walker RL. Oncogenic ets fusions deregulate E2f3 target genes in Ewing sarcoma and prostate cancer. Genome Res (2013) 23(11):1797–809. doi: 10.1101/gr.151340.112 PMC3814880 23940108

[83] Li M Chen C-W. Epigenetic and transcriptional signaling in Ewing sarcoma–disease etiology and therapeutic opportunities. Biomedicines (2022) 10(6):1325. doi: 10.3390/biomedicines10061325 35740349 PMC9219675

[84] Shi X Zheng Y Jiang L Zhou B Yang W Li L. Ews-Fli1 regulates and cooperates with core regulatory circuitry in Ewing sarcoma. Nucleic Acids Res (2020) 48(20):11434–51. doi: 10.1093/nar/gkaa901 PMC7672457 33080033

[85] Smith R Owen LA Trem DJ Wong JS Whangbo JS Golub TR. Expression profiling of Ews/Fli identifies Nkx2.2 as a critical target gene in ewing's sarcoma. Cancer Cell (2006) 9(5):405–16. doi: 10.1016/j.ccr.2006.04.004 16697960

[86] Svoboda LK Harris A Bailey NJ Schwentner R Tomazou E von Levetzow C. Overexpression of hox genes is prevalent in Ewing sarcoma and is associated with altered epigenetic regulation of developmental transcription programs. Epigenetics (2014) 9(12):1613–25. doi: 10.4161/15592294.2014.988048 PMC4622732 25625846

[87] von Heyking K Roth L Ertl M Schmidt O Calzada-Wack J Neff F. The posterior hoxd locus: Its contribution to phenotype and malignancy of Ewing sarcoma. Oncotarget (2016) 7(27):41767–80. doi: 10.18632/oncotarget.9702 PMC5173095 27363011

[88] Svoboda LK Bailey N Van Noord RA Krook MA Harris A Cramer C. Tumorigenicity of Ewing sarcoma is critically dependent on the trithorax proteins Mll1 and menin. Oncotarget (2017) 8(1):458–71. doi: 10.18632/oncotarget.13444 PMC5352134 27888797

[89] Orth MF Surdez D Marchetto A Grossetête S Gerke JS Zaidi S. Systematic multi-omics cell line profiling uncovers principles of Ewing sarcoma fusion oncogene-mediated gene regulation. bioRxiv (2021). doi: 10.1101/2021.06.08.447518 PMC10333306 36476851

[90] Wiles ET Bell R Thomas D Beckerle M Lessnick SL. Zeb2 represses the epithelial phenotype and facilitates metastasis in Ewing sarcoma. Genes Cancer (2013) 4(11-12):486–500. doi: 10.1177/1947601913506115 24386509 PMC3877663

[91] Kinsey M Smith R Iyer AK McCabe ERB Lessnick SL. Ews/Fli and its downstream target Nr0b1 interact directly to modulate transcription and oncogenesis in ewing's sarcoma. Cancer Res (2009) 69(23):9047–55. doi: 10.1158/0008-5472.Can-09-1540 PMC2789197 19920188

[92] Wiles ET Lui-Sargent B Bell R Lessnick SL. Bcl11b is up-regulated by Ews/Fli and contributes to the transformed phenotype in Ewing sarcoma. PloS One (2013) 8(3):e59369. doi: 10.1371/journal.pone.0059369 23527175 PMC3601955

[93] Beauchamp E Bulut G Abaan O Chen K Merchant A Matsui W. Gli1 is a direct transcriptional target of ews-Fli1 oncoprotein. J Biol Chem (2009) 284(14):9074–82. doi: 10.1074/jbc.M806233200 PMC2666556 19189974

[94] Toki S Wakai S Sekimizu M Mori T Ichikawa H Kawai A. Pax7 immunohistochemical evaluation of Ewing sarcoma and other small round cell tumours. Histopathology (2018) 73(4):645–52. doi: 10.1111/his.13689 29920735

[95] Charville GW Wang W-L Ingram DR Roy A Thomas D Patel RM. Ewsr1 fusion proteins mediate Pax7 expression in Ewing sarcoma. Modern Pathol (2017) 30(9):1312–20. doi: 10.1038/modpathol.2017.49 28643791

[96] Grünewald TGP Bernard V Gilardi-Hebenstreit P Raynal V Surdez D Aynaud M-M. Chimeric Ewsr1-Fli1 regulates the Ewing sarcoma susceptibility gene Egr2 *Via* a ggaa microsatellite. Nat Genet (2015) 47(9):1073–8. doi: 10.1038/ng.3363 PMC4591073 26214589

[97] Buchou C Laud-Duval K van der Ent W Grossetête S Zaidi S Gentric G. Upregulation of the mevalonate pathway through Ewsr1-Fli1/Egr2 regulatory axis confers Ewing cells exquisite sensitivity to statins. Cancers (2022) 14(9):2327. doi: 10.3390/cancers14092327 35565457 PMC9100622

[98] Yang L Hu H-M Zielinska-Kwiatkowska A Chansky HA. Foxo1 is a direct target of ews-Fli1 oncogenic fusion protein in ewing’s sarcoma cells. Biochem Biophys Res Commun (2010) 402(1):129–34. doi: 10.1016/j.bbrc.2010.09.129 PMC2981438 20933505

[99] Kim S Denny CT Wisdom R. Cooperative DNA binding with ap-1 proteins is required for transformation by ews-ets fusion proteins. Mol Cell Biol (2006) 26(7):2467–78. doi: 10.1128/MCB.26.7.2467-2478.2006 PMC1430316 16537893

[100] Cox TR. The matrix in cancer. Nat Rev Cancer (2021) 21(4):217–38. doi: 10.1038/s41568-020-00329-7 33589810

[101] Binnewies M Roberts EW Kersten K Chan V Fearon DF Merad M. Understanding the tumor immune microenvironment (Time) for effective therapy. Nat Med (2018) 24(5):541–50. doi: 10.1038/s41591-018-0014-x PMC5998822 29686425

[102] Foster DS Jones RE Ransom RC Longaker MT Norton JA. The evolving relationship of wound healing and tumor stroma. JCI Insight (2018) 3(18):e99911. doi: 10.1172/jci.insight.99911 30232274 PMC6237224

[103] Bissell MJ Hines WC. Why don't we get more cancer? a proposed role of the microenvironment in restraining cancer progression. Nat Med (2011) 17(3):320–9. doi: 10.1038/nm.2328 PMC3569482 21383745

[104] Ehnman M Chaabane W Haglund F Tsagkozis P. The tumor microenvironment of pediatric sarcoma: Mesenchymal mechanisms regulating cell migration and metastasis. Curr Oncol Rep (2019) 21(10):1–11. doi: 10.1007/s11912-019-0839-6 PMC6695368 31418125

[105] Saggioro M D'Angelo E Bisogno G Agostini M Pozzobon M. Carcinoma and sarcoma microenvironment at a glance: Where we are. Front Oncol (2020) 10:76. doi: 10.3389/fonc.2020.00076 32195166 PMC7063801

[106] Volchenboum SL Andrade J Huang L Barkauskas DA Krailo M Womer RB. Gene expression profiling of Ewing sarcoma tumors reveals the prognostic importance of tumor-stromal interactions: A report from the children's oncology group. J Pathol Clin Res (2015) 1(2):83–94. doi: 10.1002/cjp2.9 26052443 PMC4457396

[107] Applebaum MA Worch J Matthay KK Goldsby R Neuhaus J West DC. Clinical features and outcomes in patients with extraskeletal Ewing sarcoma. Cancer (2011) 117(13):3027–32. doi: 10.1002/cncr.25840 PMC3135782 21692057

[108] Saggese G Baroncelli GI Bertelloni S. Puberty and bone development. Best Pract Res Clin Endocrinol Metab (2002) 16(1):53–64. doi: 10.1053/beem.2001.0180 11987898

[109] Redini F Heymann D. Bone tumor environment as a potential therapeutic target in Ewing sarcoma. Front Oncol (2015) 5:279(279). doi: 10.3389/fonc.2015.00279 26779435 PMC4688361

[110] Salhotra A Shah HN Levi B Longaker MT. Mechanisms of bone development and repair. Nat Rev Mol Cell Biol (2020) 21(11):696–711. doi: 10.1038/s41580-020-00279-w 32901139 PMC7699981

[111] Croucher PI McDonald MM Martin TJ. Bone metastasis: The importance of the neighbourhood. Nat Rev Cancer (2016) 16(6):373–86. doi: 10.1038/nrc.2016.44 27220481

[112] Hauer K Calzada-Wack J Steiger K Grunewald TGP Baumhoer D Plehm S. Dkk2 mediates osteolysis, invasiveness, and metastatic spread in Ewing sarcoma. Cancer Res (2013) 73(2):967–77. doi: 10.1158/0008-5472.can-12-1492 23204234

[113] Todd VM Johnson RW. Hypoxia in bone metastasis and osteolysis. Cancer Lett (2020) 489:144–54. doi: 10.1016/j.canlet.2020.06.004 PMC7429356 32561416

[114] Anderson JL Titz B Akiyama R Komisopoulou E Park A Tap WD. Phosphoproteomic profiling reveals Il6-mediated paracrine signaling within the Ewing sarcoma family of tumors. Mol Cancer Res (2014) 12(12):1740–54. doi: 10.1158/1541-7786.mcr-14-0159 25092916

[115] Sharma V Srinivasan A Nikolajeff F Kumar S. Biomineralization process in hard tissues: The interaction complexity within protein and inorganic counterparts. Acta Biom (2021) 120:20–37. doi: 10.1016/j.actbio.2020.04.049 32413577

[116] Sims NA Martin TJ. Osteoclasts provide coupling signals to osteoblast lineage cells through multiple mechanisms. Annu Rev Physiol (2020) 82:507–29. doi: 10.1146/annurev-physiol-021119-034425 31553686

[117] Prideaux M Findlay DM Atkins GJ. Osteocytes: The master cells in bone remodelling. Curr Opin Pharmacol (2016) 28:24–30. doi: 10.1016/j.coph.2016.02.003 26927500

[118] Baron R Kneissel M. Wnt signaling in bone homeostasis and disease: From human mutations to treatments. Nat Med (2013) 19(2):179–92. doi: 10.1038/nm.3074 23389618

[119] Jann J Gascon S Roux S Faucheux N. Influence of the tgf-β superfamily on Osteoclasts/Osteoblasts balance in physiological and pathological bone conditions. Int J Mol Sci (2020) 21(20):7597. doi: 10.3390/ijms21207597 33066607 PMC7589189

[120] Lissat A Joerschke M Shinde DA Braunschweig T Meier A Makowska A. Il6 secreted by Ewing sarcoma tumor microenvironment confers anti-apoptotic and cell-disseminating paracrine responses in Ewing sarcoma cells. BMC Cancer (2015) 15(1):552. doi: 10.1186/s12885-015-1564-7 26215971 PMC4517368

[121] Taylor R Knowles HJ Athanasou NA. Ewing Sarcoma cells express RANKL and support osteoclastogenesis. J Pathol (2011) 225(2):195–202. doi: 10.1002/path.2869 21547906

[122] Trivedi T Pagnotti GM Guise TA Mohammad KS. The role of TGF in bone metastases. Biomolecules (2021) 11(11):1643. doi: 10.3390/biom11111643 34827641 PMC8615596

[123] Verrecchia F Rédini F. Transforming growth factor-β signaling plays a pivotal role in the interplay between osteosarcoma cells and their microenvironment. Front Oncol (2018) 8:133. doi: 10.3389/fonc.2018.00133 29761075 PMC5937053

[124] Hawkins AG Pedersen EA Treichel S Temprine K Sperring C Read JA. Wnt/β-Catenin–activated Ewing sarcoma cells promote the angiogenic switch. JCI Insight (2020) 5(13):e135188. doi: 10.1172/jci.insight.135188 32544094 PMC7406270

[125] Hawkins AG Basrur V da Veiga Leprevost F Pedersen E Sperring C Nesvizhskii AI. The Ewing sarcoma secretome and its response to activation of Wnt/Beta-catenin signaling. Mol Cell Proteomics MCP (2018) 17(5):901–12. doi: 10.1074/mcp.RA118.000596 PMC5930412 29386236

[126] Hayashi M Baker A Goldstein SD Albert CM Jackson KW McCarty G. Inhibition of porcupine prolongs metastasis free survival in a mouse xenograft model of Ewing sarcoma. Oncotarget (2017) 8(45):78265–76. doi: 10.18632/oncotarget.19432 PMC5667961 29108227

[127] Hancock JD Lessnick SL. A transcriptional profiling meta-analysis reveals a core ews-fli gene expression signature. Cell Cycle (2008) 7(2):250–6. doi: 10.4161/cc.7.2.5229 18256529

[128] Theocharis AD Skandalis SS Gialeli C Karamanos NK. Extracellular matrix structure. Adv Drug Deliv Rev (2016) 97:4–27. doi: 10.1016/j.addr.2015.11.001 26562801

[129] Cox TR Bird D Baker A-M Barker HE Ho MW Lang G. Lox-mediated collagen crosslinking is responsible for fibrosis-enhanced metastasis. Cancer Res (2013) 73(6):1721–32. doi: 10.1158/0008-5472.CAN-12-2233 PMC3672851 23345161

[130] Erler JT Bennewith KL Cox TR Lang G Bird D Koong A. Hypoxia-induced lysyl oxidase is a critical mediator of bone marrow cell recruitment to form the premetastatic niche. Cancer Cell (2009) 15(1):35–44. doi: 10.1016/j.ccr.2008.11.012 19111879 PMC3050620

[131] Langlois B Saupe F Rupp T Arnold C van der Heyden M Orend G. Angiomatrix, a signature of the tumor angiogenic switch-specific matrisome, correlates with poor prognosis for glioma and colorectal cancer patients. Oncotarget (2014) 5(21):10529–45. doi: 10.18632/oncotarget.2470 PMC4279391 25301723

[132] Rankin EB Giaccia AJ. Hypoxic control of metastasis. Science (2016) 352(6282):175–80. doi: 10.1126/science.aaf4405 PMC4898055 27124451

[133] Aryee DN Niedan S Kauer M Schwentner R Bennani-Baiti IM Ban J. Hypoxia modulates ews-Fli1 transcriptional signature and enhances the malignant properties of Ewing's sarcoma cells in vitro. Cancer Res (2010) 70(10):4015–23. doi: 10.1158/0008-5472.Can-09-4333 PMC2884367 20442286

[134] Knowles HJ Schaefer K-L Dirksen U Athanasou NA. Hypoxia and hypoglycaemia in ewing's sarcoma and osteosarcoma: Regulation and phenotypic effects of hypoxia-inducible factor. BMC Cancer (2010) 10(1):372. doi: 10.1186/1471-2407-10-372 20637078 PMC2918574

[135] Lu C Mahajan A Hong S-H Galli S Zhu S Tilan JU. Hypoxia-activated neuropeptide Y/Y5 Receptor/Rhoa pathway triggers chromosomal instability and bone metastasis in Ewing sarcoma. Nat Commun (2022) 13(1):2323. doi: 10.1038/s41467-022-29898-x 35484119 PMC9051212

[136] Vaupel P Multhoff G. Revisiting the Warburg effect: Historical dogma versus current understanding. J Physiol (2021) 599(6):1745–57. doi: 10.1113/JP278810 33347611

[137] Kierans S Taylor C. Regulation of glycolysis by the hypoxia-inducible factor (Hif): Implications for cellular physiology. J Physiol (2021) 599(1):23–37. doi: 10.1113/JP280572 33006160

[138] Dasgupta A Trucco M Rainusso N Bernardi RJ Shuck R Kurenbekova L. Metabolic modulation of Ewing sarcoma cells inhibits tumor growth and stem cell properties. Oncotarget (2017) 8(44):77292–308. doi: 10.18632/oncotarget.20467 PMC5652780 29100387

[139] Tanner JM Bensard C Wei P Krah NM Schell JC Gardiner J. Ews/Fli is a master regulator of metabolic reprogramming in Ewing sarcoma. Mol Cancer Res MCR (2017) 15(11):1517–30. doi: 10.1158/1541-7786.Mcr-17-0182 PMC5668171 28720588

[140] Sen N Cross AM Lorenzi PL Khan J Gryder BE Kim S. Ews-Fli1 reprograms the metabolism of Ewing sarcoma cells *Via* positive regulation of glutamine import and serine-glycine biosynthesis. Mol Carcinog (2018) 57(10):1342–57. doi: 10.1002/mc.22849 PMC6175245 29873416

[141] Issaq SH Mendoza A Kidner R Rosales TI Duveau DY Heske CM. EWS-FLI1-Regulated serine synthesis and exogenous serine are necessary for Ewing sarcoma cellular proliferation and tumor growth. Mol Cancer Ther (2020) 19(7):1520–9. doi: 10.1158/1535-7163.Mct-19-0748 PMC7335326 32371575

[142] Jiménez JA Apfelbaum AA Hawkins AG Svoboda LK Kumar A Ruiz RO. Ews-Fli1 and menin converge to regulate ATF4 activity in Ewing sarcoma. Mol Cancer Res MCR (2021) 19(7):1182–95. doi: 10.1158/1541-7786.mcr-20-0679 PMC8462528 33741715

[143] Grohar PJ Woldemichael GM Griffin LB Mendoza A Chen QR Yeung C. Identification of an inhibitor of the ews-Fli1 oncogenic transcription factor by high-throughput screening. J Natl Cancer Instit (2011) 103(12):962–78. doi: 10.1093/jnci/djr156 PMC3119649 21653923

[144] Stegmaier K Wong JS Ross KN Chow KT Peck D Wright RD. Signature-based small molecule screening identifies cytosine arabinoside as an Ews/Fli modulator in Ewing sarcoma. PloS Med (2007) 4(4):e122. doi: 10.1371/journal.pmed.0040122 17425403 PMC1851624

[145] Grohar PJ Griffin LB Yeung C Chen QR Pommier Y Khanna C. Ecteinascidin 743 interferes with the activity of ews-Fli1 in Ewing sarcoma cells. Neoplasia (2011) 13(2):145–53. doi: 10.1593/neo.101202 PMC3033593 21403840

[146] Mateo-Lozano S Tirado OM Notario V. Rapamycin induces the fusion-type independent downregulation of the Ews/Fli-1 proteins and inhibits ewing's sarcoma cell proliferation. Oncogene (2003) 22(58):9282–7. doi: 10.1038/sj.onc.1207081 14681687

[147] Zollner SK Selvanathan SP Graham GT Commins RMT Hong SH Moseley E. Inhibition of the oncogenic fusion protein ews-Fli1 causes G2-m cell cycle arrest and enhanced vincristine sensitivity in ewing's sarcoma. Sci Signal (2017) 10(499):eaam8429. doi: 10.1126/scisignal.aam8429 28974650 PMC7330879

[148] Grohar PJ Segars LE Yeung C Pommier Y D'Incalci M Mendoza A. Dual targeting of ews-Fli1 activity and the associated DNA damage response with trabectedin and Sn38 synergistically inhibits Ewing sarcoma cell growth. Clin Cancer Res (2014) 20(5):1190–203. doi: 10.1158/1078-0432.Ccr-13-0901 PMC5510643 24277455

[149] Ma Y Baltezor M Rajewski L Crow J Samuel G Staggs VS. Targeted inhibition of histone deacetylase leads to suppression of Ewing sarcoma tumor growth through an unappreciated ews-Fli1/Hdac3/Hsp90 signaling axis. J Mol Med (2019) 97(7):957–72. doi: 10.1007/s00109-019-01782-0 PMC6584050 31025088

[150] Loganathan SN Tang N Fleming JT Ma Y Guo Y Borinstein SC. Bet bromodomain inhibitors suppress EWS-FLI1-Dependent transcription and the IFG1 autocrine mechanism in Ewing sarcoma. Oncotarget (2016) 7(28):43504–17. doi: 10.18632/oncotarget.9762 PMC5190040 27259270

[151] Hensel T Giorgi C Schmidt O Calzada-Wack J Neff F Buch T. Targeting the EWS-ETS transcriptional program by BET bromodomain inhibition in Ewing sarcoma. Oncotarget (2016) 7(2):1451–63. doi: 10.18632/oncotarget.6385 PMC4811472 26623725

[152] Jacques C Lamoureux F Baud'huin M Rodriguez Calleja L Quillard T Amiaud J. Targeting the epigenetic readers in Ewing sarcoma inhibits the oncogenic transcription factor Ews/Fli1. Oncotarget (2016) 7(17):24125–40. doi: 10.18632/oncotarget.8214 PMC5029689 27006472

[153] Parrish JK McCann TS Sechler M Sobral LM Ren W Jones KL. The jumonji-domain histone demethylase inhibitor jib-04 deregulates oncogenic programs and increases DNA damage in Ewing sarcoma, resulting in impaired cell proliferation and survival, and reduced tumor growth. Oncotarget (2018) 9(69):33110–23. doi: 10.18632/oncotarget.26011 PMC6145692 30237855

[154] Jaboin J Wild J Hamidi H Khanna C Kim CJ Robey R. Ms-27-275, an inhibitor of histone deacetylase, has marked in vitro and in vivo antitumor activity against pediatric solid tumors. Cancer Res (2002) 62(21):6108–15. 12414635

[155] Pedot G Marques JG Ambühl PP Wachtel M Kasper S Ngo QA. Inhibition of hdacs reduces Ewing sarcoma tumor growth through ews-Fli1 protein destabilization. Neoplasia (2022) 27:100784. doi: 10.1016/j.neo.2022.100784 35366465 PMC8971315

[156] Schmidt O Nehls N Prexler C von Heyking K Groll T Pardon K. Class I histone deacetylases (Hdac) critically contribute to Ewing sarcoma pathogenesis. J Exp Clin Cancer Res (2021) 40(1):322. doi: 10.1186/s13046-021-02125-z 34654445 PMC8518288

[157] Gröbner SN Worst BC Weischenfeldt J Buchhalter I Kleinheinz K Rudneva VA. The landscape of genomic alterations across childhood cancers. Nature (2018) 555(7696):321–7. doi: 10.1038/nature25480 29489754

[158] Cillo AR Mukherjee E Bailey NG Onkar S Daley J Salgado C. Ewing Sarcoma and osteosarcoma have distinct immune signatures and intercellular communication networks. Clin Cancer Res (2022) OF1–OF15. doi: 10.1158/1078-0432.Ccr-22-1471 PMC9669190 36074145

[159] Machado I López-Guerrero JA Scotlandi K Picci P Llombart-Bosch A. Immunohistochemical analysis and prognostic significance of pd-L1, pd-1, and Cd8+ tumor-infiltrating lymphocytes in ewing’s sarcoma family of tumors (Esft). Virchows Archiv (2018) 472(5):815–24. doi: 10.1007/s00428-018-2316-2 29445891

[160] Morales E Olson M Iglesias F Dahiya S Luetkens T Atanackovic D. Role of immunotherapy in Ewing sarcoma. J Immunother Cancer (2020) 8(2):e000653. doi: 10.1136/jitc-2020-000653 33293354 PMC7725096

[161] Berghuis D de Hooge AS Santos SJ Horst D Wiertz EJ van Eggermond MC. Reduced human leukocyte antigen expression in advanced-stage Ewing sarcoma: Implications for immune recognition. J Pathol (2009) 218(2):222–31. doi: 10.1002/path.2537 19274709

[162] Terry RL Meyran D Fleuren EDG Mayoh C Zhu J Omer N. Chimeric antigen receptor T cell therapy and the immunosuppressive tumor microenvironment in pediatric sarcoma. Cancers (2021) 13(18):4704. doi: 10.3390/cancers13184704 34572932 PMC8465026

[163] Bailey KM Julian CM Klinghoffer AN Bernard H Lucas PC McAllister-Lucas LM. Ews-Fli1 low Ewing sarcoma cells demonstrate decreased susceptibility to T-Cell-Mediated tumor cell apoptosis. Oncotarget (2019) 10(36):3385–99. doi: 10.18632/oncotarget.26939 PMC6534359 31164960

[164] Zhang H Maric I DiPrima MJ Khan J Orentas RJ Kaplan RN. Fibrocytes represent a novel mdsc subset circulating in patients with metastatic cancer. Blood (2013) 122(7):1105–13. doi: 10.1182/blood-2012-08-449413 PMC3744987 23757729

[165] Wang Y Ding Y Guo N Wang S. Mdscs: Key criminals of tumor pre-metastatic niche formation. Front Immunol (2019) 10:172. doi: 10.3389/fimmu.2019.00172 30792719 PMC6374299

[166] Sangaletti S Tripodo C Santangelo A Castioni N Portararo P Gulino A. Mesenchymal transition of high-grade breast carcinomas depends on extracellular matrix control of myeloid suppressor cell activity. Cell Rep (2016) 17(1):233–48. doi: 10.1016/j.celrep.2016.08.075 27681434

[167] King RJ Shukla SK He C Vernucci E Thakur R Attri KS. Cd73 induces gm-Csf/Mdsc-Mediated suppression of T cells to accelerate pancreatic cancer pathogenesis. Oncogene (2022) 41(7):971–82. doi: 10.1038/s41388-021-02132-6 PMC8840971 35001076

[168] Walsh NC Kenney LL Jangalwe S Aryee KE Greiner DL Brehm MA. Humanized mouse models of clinical disease. Annu Rev Pathol (2017) 12:187–215. doi: 10.1146/annurev-pathol-052016-100332 27959627 PMC5280554

[169] Vasileva E Warren M Triche TJ Amatruda JF. Dysregulated heparan sulfate proteoglycan metabolism promotes Ewing sarcoma tumor growth. eLife (2022) 11:e69734. doi: 10.7554/eLife.69734 35285802 PMC8942468

[170] Zöllner SK Amatruda JF Bauer S Collaud S de Álava E DuBois SG. Ewing Sarcoma–diagnosis, treatment, clinical challenges and future perspectives. J Clin Med (2021) 10(8):1685. doi: 10.3390/jcm10081685 33919988 PMC8071040

[171] Barber-Rotenberg JS Selvanathan SP Kong Y Erkizan HV Snyder TM Hong SP. Single enantiomer of yk-4-279 demonstrates specificity in targeting the oncogene ews-Fli1. Oncotarget (2012) 3(2):172–82. doi: 10.18632/oncotarget.454 PMC3326647 22383402

[172] Gangwal K Sankar S Hollenhorst PC Kinsey M Haroldsen SC Shah AA. Microsatellites as Ews/Fli response elements in ewing's sarcoma. Proc Natl Acad Sci USA (2008) 105(29):10149–54. doi: 10.1073/pnas.0801073105 PMC2481306 18626011

[173] Baldauf MC Orth MF Dallmayer M Marchetto A Gerke JS Rubio RA. Robust diagnosis of Ewing sarcoma by immunohistochemical detection of super-Enhancer-Driven Ewsr1-ets targets. Oncotarget (2018) 9(2):1587–601. doi: 10.18632/oncotarget.20098 PMC5788584 29416716

[174] Joo J Christensen L Warner K States L Kang HG Vo K. Gli1 is a central mediator of Ews/Fli1 signaling in Ewing tumors. PloS One (2009) 4(10):e7608. doi: 10.1371/journal.pone.0007608 19859563 PMC2763206

[175] Niedan S Kauer M Aryee DNT Kofler R Schwentner R Meier A. Suppression of Foxo1 is responsible for a growth regulatory repressive transcriptional Sub-signature of ews-Fli1 in Ewing sarcoma. Oncogene (2014) 33(30):3927–38. doi: 10.1038/onc.2013.361 PMC4114138 23995784

